# Genome-Wide Association Study of Root Mealiness and Other Texture-Associated Traits in Cassava

**DOI:** 10.3389/fpls.2021.770434

**Published:** 2021-12-17

**Authors:** Kelechi Uchendu, Damian Ndubuisi Njoku, Agre Paterne, Ismail Yusuf Rabbi, Daniel Dzidzienyo, Pangirayi Tongoona, Samuel Offei, Chiedozie Egesi

**Affiliations:** ^1^West Africa Centre for Crop Improvement (WACCI), University of Ghana, Accra, Ghana; ^2^National Root Crops Research Institute (NRCRI), Umudike, Nigeria; ^3^International Institute of Tropical Agriculture (IITA), Ibadan, Nigeria; ^4^Department of Plant Breeding and Genetics, Cornell University, Ithaca, NY, United States

**Keywords:** *Manihot esculenta* Crantz, SNP markers, genome-wide association study, root mealiness, marker-assisted selection, culinary qualities

## Abstract

Cassava breeders have made significant progress in developing new genotypes with improved agronomic characteristics such as improved root yield and resistance against biotic and abiotic stresses. However, these new and improved cassava (*Manihot esculenta* Crantz) varieties in cultivation in Nigeria have undergone little or no improvement in their culinary qualities; hence, there is a paucity of genetic information regarding the texture of boiled cassava, particularly with respect to its mealiness, the principal sensory quality attribute of boiled cassava roots. The current study aimed at identifying genomic regions and polymorphisms associated with natural variation for root mealiness and other texture-related attributes of boiled cassava roots, which includes fibre, adhesiveness (ADH), taste, aroma, colour, and firmness. We performed a genome-wide association (GWAS) analysis using phenotypic data from a panel of 142 accessions obtained from the National Root Crops Research Institute (NRCRI), Umudike, Nigeria, and a set of 59,792 high-quality single nucleotide polymorphisms (SNPs) distributed across the cassava genome. Through genome-wide association mapping, we identified 80 SNPs that were significantly associated with root mealiness, fibre, adhesiveness, taste, aroma, colour and firmness on chromosomes 1, 4, 5, 6, 10, 13, 17 and 18. We also identified relevant candidate genes that are co-located with peak SNPs linked to these traits in *M. esculenta*. A survey of the cassava reference genome v6.1 positioned the SNPs on chromosome 13 in the vicinity of *Manes*.*13G026900*, a gene recognized as being responsible for cell adhesion and for the mealiness or crispness of vegetables and fruits, and also known to play an important role in cooked potato texture. This study provides the first insights into understanding the underlying genetic basis of boiled cassava root texture. After validation, the markers and candidate genes identified in this novel work could provide important genomic resources for use in marker-assisted selection (MAS) and genomic selection (GS) to accelerate genetic improvement of root mealiness and other culinary qualities in cassava breeding programmes in West Africa, especially in Nigeria, where the consumption of boiled and pounded cassava is low.

## Introduction

The texture of boiled cassava roots is an important organoleptic attribute and criterion for the acceptance of new improved genotypes by end-users. Thus, quality assessment of texture in boiled roots is a crucial step in cassava breeding. Regarding texture, root mealiness is generally cited by end-users as the principal quality attribute of boiled cassava roots (Favaro et al., 2008; Hongbété et al., 2011). Root mealiness is a complex textural trait and refers to the feel of the boiled cassava roots in the mouth. It is an important sensory attribute used in describing cassava storage roots which, when boiled in water, become soft and chewable (Ngeve, 2003). Nonetheless, mealiness of boiled cassava roots has received little research attention compared to boiled potato, partly due to major focus on improved yield and disease resistance by cassava breeders. Additionally, most of the cassava produced in sub-Saharan Africa (SSA) and Nigeria in particular are processed into marketable granulated and paste products (Nweke et al., 2002). Consequently, improving cooking and eating quality traits, predominantly mealiness, are major goals for cassava breeding programmes to address the demand for varieties that are suitable for the fresh consumption market segment.

Root mealiness is difficult to phenotype because of the individual and complex nature of textural perception. Assessment of this trait depends on both subjective estimates (also referred to as “sensory evaluation”) and semi-throughput methods such as instrumental techniques that correlate well with scores from sensory panels. Sensory evaluation refers to descriptive test analysis carried out by a trained subjective taste panel. Instrumental firmness analysis (IFA) is an instrumental method in which the cone of a penetrometer is pushed to a penetration depth of 1 cm into boiled cassava root sections and the peak force of penetration recorded (Iragaba et al., 2019). Though sensory analysis is still the final arbiter of cassava cooking and eating quality, there are some setbacks in its application in cassava breeding. It can be time consuming, laborious, costly, low throughput, and only a few samples (*n* < 15) can be evaluated per day, restricting assessment of root mealiness and other textural quality traits to later stages in the selection cycle of a cassava breeding programme. Furthermore, cassava has a lengthy breeding cycle due to its inherent heterozygosity, poor and asynchronous flowering, insufficient seed production, slow multiplication rate of planting materials, and a long annual growing cycle of 12 months (Ceballos et al., 2015). These challenges make conventional breeding approaches rigid and inefficient, with a low level of genetic gain. Understanding the genetic basis of variation in root mealiness and other texture-related traits is critical for increasing their selection efficiency, shortening the breeding cycle and the rate of genetic gain.

Genetic studies on texture, especially putative candidate genes and quantitative trait loci (QTL) influencing textural properties, are widely established in cooked rice and potato tuber crops but rather limited for boiled cassava roots. For instance, using single locus and multi-locus GWAS approaches, Misra et al. (2018) detected multiple major/minor quantitative trait nucleotides (QTNs) associated with rice cooking properties. Further, they found hot spot QTLs on chromosome 6 where QTNs for adhesiveness co-localized with QTNs for amylose content. A 2011 study identified QTL for cooked potato tuber texture in a diploid biparental mapping population (Lloyd, 2011). Interestingly, one of the QTLs detected occurred close to a pectin methyl esterase (PME) gene (Lloyd, 2011).

The availability of cassava genomic resources has increased significantly in recent years, especially through the sequencing of the cassava reference genome (Prochnik et al., 2012), multi-parental SNP-based molecular linkage map (ICGMC, 2015), high-density genotyping using next-generation sequencing (Rabbi et al., 2014), and cassava haplotype map of segregating variants from deep sequencing of hundreds of different accessions (Ramu et al., 2017). With these increased resources, it is now feasible to examine the genetic architecture of boiled cassava root texture. To date, several genome-wide association mapping studies have been applied successfully to identify QTL or candidate genes associated with useful agronomic and quality-related traits in cassava at the whole-genome level, including cassava mosaic disease resistance (Wolfe et al., 2016), resistance to cassava green mite pest (Ezenwaka et al., 2018; Jiwuba et al., 2020), cassava brown streak disease resistance (Kayondo et al., 2018), dry matter and provitamin A carotenoid content (Esuma et al., 2016; Rabbi et al., 2017; Ikeogu et al., 2019) and shoot weight, fresh root yield, amylose content, dry matter content, and starch yield (de Oliveira et al., 2012). However, a genome-wide association study (GWAS) for root mealiness and other texture-related traits (softness, firmness, adhesiveness, fibre, taste, aroma, and colour) has not yet been reported in *Manihot esculenta*. Accordingly, the aim of this present study was to identify genomic regions and single nucleotide polymorphisms (SNPs) associated with natural variations for root mealiness and other textural quality traits in cassava.

## Materials and Methods

### Plant Material and Field Trials

An association mapping panel of 150 cassava accessions sourced from the National Root Crops Research Institute (NRCRI) in Umudike, Nigeria, was used in this study (Supplementary Table S1). These accessions showed variability in storage root flesh colour ranging from white to deep yellow.

The accessions were planted in two field growing seasons (2017–2018 and 2018–2019) across three contrasting cassava growing agro-ecological zones of Nigeria: Umudike (mean annual rainfall of 2,200 mm; mean annual temperature of 22 to 31°C; coordinates 5° 29′ N, 7° 24′ E; altitude of 120 m above sea level; dystric-luvisol soils; humid forest zone); Igbariam (mean annual rainfall of 1,800 mm; mean annual temperature of 24 to 32°C; coordinates 5° 56′ N, 7° 31′ E; altitude of 150 m above sea level; dystric-luvisol soils; forest savannah transition zone); and Otobi (mean annual rainfall of 1,500 mm; mean annual temperature of 24 to 35°C; coordinates 7° 20′ N, 8° 41′ E; altitude of 319 m above sea level; ferric-luvisol soils; derived savannah zone).

Field trials were established at the onset of rains using 15 × 10 alpha lattice design with three replications in each location. A replication consisted of 10 randomized incomplete blocks each containing 15 accessions. Accessions were planted as single rows of 5 plants with inter-row spacing of 1 m and intra-row spacing of 0.8 m, making a basic plot size of 4 m^2^. Blocks were separated by 1.2 m alleys. No fertilizers or herbicides were applied; however, fields were kept clean by regular hand weeding. Trials were harvested 12 months after planting (MAP).

### Phenotyping

The association panel of 150 accessions was phenotyped for root mealiness and other important quality attributes of boiled cassava roots (taste, softness, fibre, adhesiveness, aroma, and colour) using subjective estimates. Firmness of boiled cassava roots was determined using a puncture force test.

#### Boiled Cassava Sample Preparation

Freshly harvested and healthy cassava roots were selected from each genotype, peeled and cut with a kitchen knife into roughly uniform-sized pieces. Samples for sensory analysis (10 root pieces) were washed twice, immersed in boiling water in enamel pots on a domestic gas cooker and left to cook for a fixed period of 25 min. The labelled boiled root samples were then removed from the pots and kept warm in an insulated box until ready to be served for sensory analysis.

#### Descriptive Sensory Analysis

A panel of 10 assessors participated in the descriptive sensory test and evaluated the boiled root samples during the two consecutive years. These assessors were indigenes of the major cassava growing communities in Nigeria who regularly use cassava in their diets. They were recruited based on their interest and availability to participate. Mealiness, taste, softness, fibre, adhesiveness, aroma, and colour were the quality attributes of boiled cassava considered in this study. The assessors were thus trained to understand and quantify these attributes using numeric ratings based on hedonic scales, as described in Raji et al. (2007) with slight modification (Table 1). The attributes were scored after assessors tasted the samples. Ten samples (accessions) were evaluated per session. Samples were presented to assessors in white plastic plates at room temperature, coded with random three-digit numbers. Boiled root samples were consumed plain. Water was provided to the assessors for mouth rinsing before and between tasting samples. Assessors performed the sensory test independently in separate tasting booths, with no interaction among assessors.

**Table 1:** Scales used in sensory analysis of boiled cassava roots.

Organoleptic attribute^a^	Descriptor
Mealiness	0: Non-mealy; 1: Fairly mealy; 2: Mealy; 3: Very mealy
Fibre	1: Low fibre; 2: Moderate fibre; 3: High fibre
Adhesiveness	1: Non-sticky; 2: Slightly sticky; 3: Sticky
Softness	1: Very hard; 2: Hard; 3: Soft; 4: Very soft
Taste	1: Bitter; 2: Bland; 3: Sweet
Colour	1: Yellow; 2: Cream; 3: White
Aroma	1: Unpleasant; 2: Bland; 3: Pleasant

a *Hedonic scale: mealiness (0–3); fibre (1–3); adhesiveness (1–3); softness (1–4); taste (1–3); colour (1–3); aroma (1–3)*.

#### Instrumental Firmness Analysis

Instrumental test was performed using a digital penetrometer (Model number: FHP-803, Vetus Industrial Company Limited, Hefei, China) to assess the firmness of boiled cassava roots. Three roots of each accession were peeled, washed and cut into 3-cm-thick slices using a kitchen knife and ruler. For each accession, three slices per root were randomly selected, immersed in boiling water in enamel pots on a domestic gas cooker and boiled for a period of 25 min. Firmness was assessed by pushing the 7.9 mm diameter tip of the penetrometer to a final penetration depth of 1 cm into each boiled root slice. Firmness was defined as the peak force of penetration reached during the test. Two measurements were made on each sliced root. Each result was expressed as the mean value (in kg) of 18 readings.

### Genotyping

#### Genomic DNA Preparation

Approximately 1 g of young fresh leaf samples from mature cassava plants in the field were collected for each accession. The leaf tissues were placed in well labelled extraction tubes arranged in a labelled 96-well box and kept on ice to maintain DNA integrity before transferring to the NRCRI molecular laboratory for freeze drying at −80°C. In order to extract deoxyribonucleic acid (DNA) from tissue cells, stored leaf samples were lyophilized for 24 to 48 h and ground to a fine powder using a TissueLyzer running with a 1× speed at a rate of 1,500 strokes/min. Genomic DNA was extracted from freeze-dried leaf samples using the DNeasy 96 Plant Mini Extraction Kit (Qiagen) procedure with slight modification. The concentration and purity of the extracted DNA samples were carefully checked using a NanoDrop 1000 spectrophotometer (Thermo Fisher Scientific, Waltham, MA, United States) and agarose gel electrophoresis, respectively. The DNA samples were transferred to the Institute of Genomic Diversity, Cornell University, Ithaca, New York, United States for SNP genotyping.

#### SNP Genotyping Assay and SNP Filtering

Genotyping-by-sequencing (GBS) approach as described by Elshire et al. (2011) was used for SNP genotyping using restriction enzyme ApeKI recommended by Hamblin and Rabbi (2014). GBS was performed using the Illumina HiSeq2000. The sequenced reads were assigned to accessions using unique barcode sequences and aligned to the *Manihot esculenta* reference genome, version 6.1^1^ (ICGMC, 2015). The SNPs were called using the TASSEL 5.0 GBS pipeline version 2 (Glaubitz et al., 2014). Extracted raw dataset of 96,697 bi-allelic SNP markers was filtered using the PLINK 1.9 software (Purcell et al., 2007; Chang et al., 2015). Eight of the 150 accessions with missing data for more than 10% of the SNP markers were not included in the association analysis. SNPs with missing call rate >10%, heterozygosity >20%, and minor allele frequency (MAF) <5% in the remaining 142 accessions were removed from the genotype dataset. Also, markers with unknown or multiple chromosomes locations were removed. After the filtering and data quality control process, a total of 59,792 SNPs distributed across the 18 cassava chromosomes were thus retained and used for population structure and genomic kinship estimation, and GWAS analysis.

### Statistical Analyses

Out of the 150 accessions initially considered in this study, 142 individuals had both phenotypic and genotypic data and were used for subsequent data analyses.

#### Phenotype Data Analysis

A PROC UNIVARIATE histogram/normal plot in SAS 9.4 was used to test whether the data were normally distributed. A mixed linear model (MLM) fitted across environments was used to perform analysis of variance (ANOVA) using the *lme4* package of the R software (Vazquez et al., 2010; R Core Team, 2014). The following model was adopted:


$$Y_{ijk}=\mu +\beta_{i}+R_{ij}+G_{k}+\left(\beta_{i}\times G_{k}\right)+e_{ijkm}$$

where *Y*_ijk_ is the phenotypic value; *μ* is the grand mean; *β_i_* is the effect of environment *i*; *R_ij_* is the effect of block *j* in environment *i*; *G_k_* is the effect of genotype *k*; (*β_i_* × *G_k_*) is the genotype × environment interaction effect associated with environment *i* and genotype *k*; and *e_ijkm_* is the residual. The Best linear unbiased prediction (BLUP) values obtained from the MLM were used as phenotypes for association analysis.

#### Population Structure Analysis

Population structure of the 142 accessions was assessed using ADMIXTURE model-based clustering approach (Alexander et al., 2009) that uses a Bayesian information criterion (BIC) to assign genotypes to putative populations (K). The K-means analysis was used to infer the optimal number of clusters after varying the possible number of clusters from 2 to 40. A discriminant analysis of principal components (DAPC) was performed on the clusters identified using the first 40 principal components in the adegenet R package (Jombart et al., 2010). A cut-off value of 40% suggested through the DAPC was used to estimate the membership probabilities of the individuals for the different groups. Pairwise F-statistic (F_ST_) was computed using the VCFtools (Danecek, 2011) to estimate the genetic diversity and the associations among the different DAPC groups formed. In addition to the DAPC, neighbour-joining (NJ) phylogenetic tree was constructed using the analyses of phylogenetics and evolution (ape) R package (Paradis et al., 2004) and results from different methods were compared to infer population structure.

#### Linkage Disequilibrium Analysis

The genome-wide linkage disequilibrium (LD), which is the non-random association of alleles at different loci in a given population, was estimated using the squared correlation coefficients (*r*^2^) between the pairs of markers using R software v3.0.3 (R Core Team, 2014). Marker pairs in perfect LD (*r*^2^ = 1) were removed prior to the LD analysis. The values of *r*^2^ were plotted against genetic distance. Scatter plots and fitted smooth curves for estimating genome-wide LD decay of the values of *r*^2^ vs. the corresponding physical distances between the SNP pairs were plotted using a base scatter plot function of R software v3.0.3, “scatter.smooth” (R Core Team, 2014).

#### Genotype–Phenotype Association Analysis

In this study, association analysis for each trait was performed using the compressed mixed linear model (CMLM) method implemented in the GAPIT (Genome Association and Prediction Integrated tools) R package (Lipka et al., 2012), where the population structure (Q), kinship (K) matrix and other hidden confounding factors were fitted to control false-positive associations (Zhang et al., 2010). Variance–covariance kinship or relatedness (K) matrix was calculated using the VanRaden method (VanRaden, 2008). The genome-wide Bonferroni correction (0.05/*n*, *n* = number of SNPs used) criterion showed a stringent threshold of significance; therefore, a GWAS threshold of −log(P) = 4 was used in declaring significant marker-trait associations (MTAs), which was determined based on the quantile-quantile (Q-Q) plots and distribution of values of *p* for all the traits (Gao et al., 2016; Mogga et al., 2018; Sukumaran et al., 2018; Adewale et al., 2020). Furthermore, CMLM results were visualized from Manhattan and Q-Q plots generated using the CMplot package of R software (LiLin-Yin, 2019).

#### Candidate Gene Analysis

Candidate gene analysis was performed using significant SNPs selected from the GWAS results. Mapping of these selected SNP markers onto genes was done using the SNP location and gene description from the *M*.*esculenta*_305_v6.1.gene.gff3 file of the cassava reference genome available in Phytozome v12.1 (Goodstein et al., 2012; Batra et al., 2014), and the intersect function from bedtools (Quinlan and Hall, 2010). The gene ontology annotation was done using Panther.^2^

## Results

### Phenotypic Distribution of Traits

**Figure 1:**
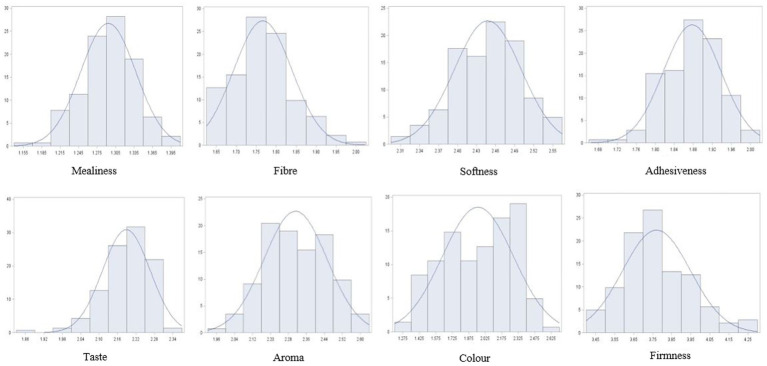
Histogram showing phenotypic distribution of cooking quality traits for 142 cassava accessions evaluated in six environments in Nigeria.

An analysis of phenotypes of the association panel averaged across the six contrasting environments showed that most traits followed a normal distribution. A few traits were slightly skewed towards the tails, particularly the distribution of fibre, taste, and firmness (Figure 1).

### Distribution of SNPs and Genetic Diversity

The genotype dataset of 96,697 SNP markers was generated using GBS (Elshire et al., 2011). A total of 59,792 genome-wide SNP markers were called for the 142 individuals after quality filtering for missing data >10%, heterozygosity >20% and MAF <5%. These SNPs were unequally distributed along the 18 chromosomes of the cassava genome sequence v6.1. SNPs coverage per chromosome varied from 2,227 on chromosome 17 to 7,382 on chromosome 1 (Supplementary Figure S1). The overall SNP diversity in the association panel was expressed by a polymorphic information content (PIC) value (Supplementary Table S2). The PIC values estimated for 59,792 SNP markers ranged from 0.09 to 0.38, with an average of 0.26. Observed heterozygosity ranged from 0.01 to 0.97, with an average of 0.31 whereas the expected heterozygosity varied from 0.10 to 0.50, with an average of 0.32 across the SNP markers. The mean minor allele frequency was 0.23 with minimum and maximum frequencies of 0.05 and 0.50, respectively. Gene diversity ranged from 0.10 to 0.50, with a mean of 0.32 (Supplementary Table S2).

### Population Structure and LD Decay

**Figure 2:**
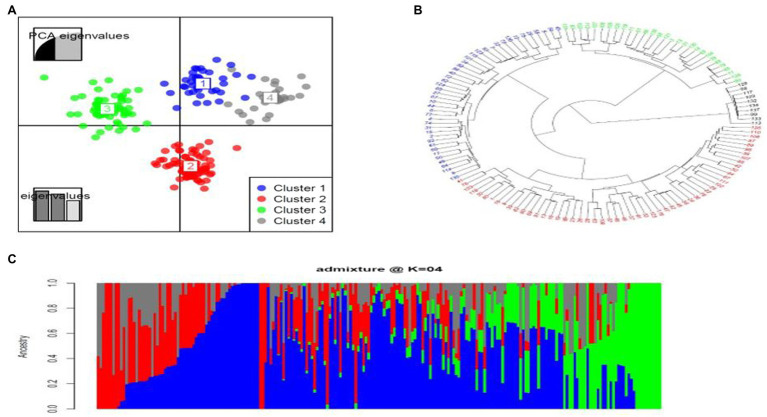
Population structure based on analysis of 59,792 SNPs. **(A)** Discriminant analysis of principal components (DAPC) using 59,792 SNP markers. The axes represent the first two Linear Discriminant (LD). Each colour represents a cluster, and each dot represents an individual. Numbers represent the different subpopulations identified by DAPC analysis. **(B)** Neighbor-joining (NJ) phylogenetic tree displaying the genetic relationships among the 142 cassava accessions in the panel based on 59,792 SNP markers. **(C)** Admixture plot showing clustering of 142 cassava accessions into clusters based on the molecular data using Bayesian-based clustering analysis. Each accession is represented by a vertical bar. The coloured sections within each bar indicate membership coefficients of the accessions in the different subgroups.

Population structure is required in genome-wide association (GWAS) analysis in order to avoid false-positive associations (Yu et al., 2006). Admixture was used for population structure analysis. The population stratification inferred by using admixture model-based clustering method indicated the presence of four subgroups (Figure 2C) and this was consistent with the number of clusters identified using DAPC analysis (Figure 2A). Estimation of the cluster membership showed that cluster two had the highest number of accessions (58), followed by cluster three with 35 accessions, cluster one with 28 accessions and cluster four with the least number of accessions (21; Figure 2A). The results of the neighbour-joining (NJ) phylogenetic tree also showed four subgroups (Figure 2B).

**Figure 3:**
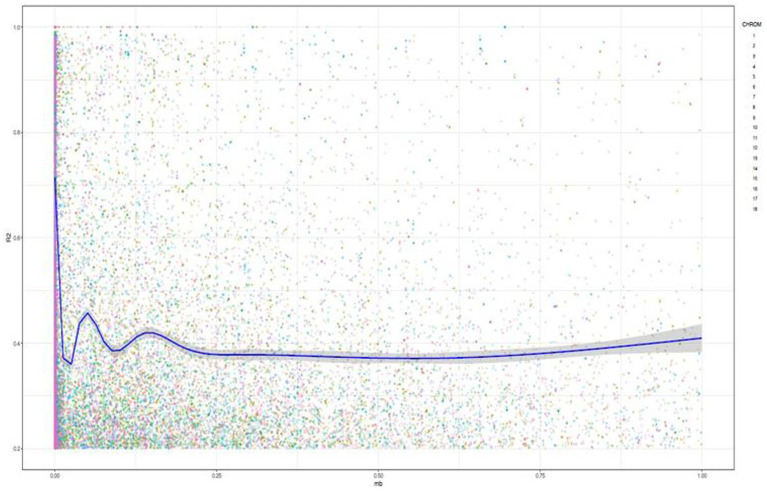
Genome-wide linkage disequilibrium (LD) decay among the SNP pairs as a function of genetic distance in base pairs based on the joint analysis of the 18 chromosomes in 142 cassava accessions. The dots correspond to the observed LD (*r*^2^) values. The blue line represents the nonlinear trend of expected LD decay.

Linkage disequilibrium analysis revealed the presence of 225,072 loci pairs within a physical distance extending up to 8821.95 bp. Pearson’s correlation coefficients showed low and positive correlation (*r* = 0.028) between the linkage disequilibrium (*R*^2^) and the physical distance (bp) as well as between the value of *p* and *R*^2^ (*r* = 0.35), indicating the existence of linkage decay (Figure 3). Assessment of the LD plotting indicated significant drop of the *r^2^* value across all the chromosomes at 0.03 Mb.

### SNP Markers Associated With Textural Attributes

Eight textural attributes of boiled cassava roots were considered for association analysis, mealiness, softness, adhesiveness, fibre, taste, aroma, colour and firmness. However, all the GWAS analysis for softness showed non-significant marker-trait association (MTA) for the panel of SNPs used in this study. Therefore, only association results for the remaining seven attributes are shown in this study. A total of 80 significant SNPs associated with textural attributes of boiled cassava roots were detected at a GWAS threshold of −log(P) = 4 (Supplementary Table S3). GWAS analysis using compressed MLM identified 7, 1, 62, 5, 1, 3 and 1 significant SNP markers associated with variation in mealiness, fibre, adhesiveness, taste, aroma, colour and firmness, respectively.

#### Root Mealiness

**Figure 4:**
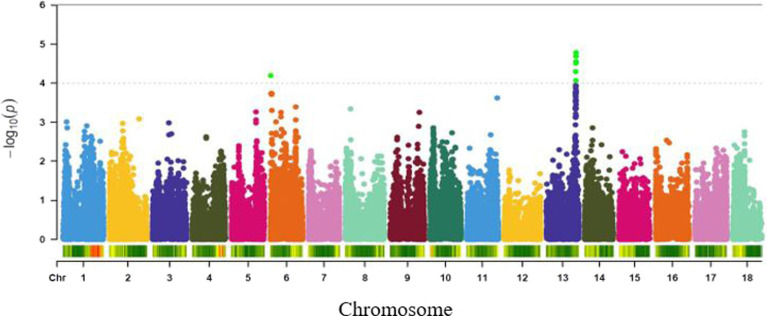
Manhattan plot for GWAS based on best linear unbiased predictions for root mealiness. The dashed horizontal line depicts the genome-wide significance threshold.

Genome-wide association study for variation in mealiness of boiled roots revealed two genomic regions that were significantly associated with the trait (Figure 4). The most significant locus occurred on chromosome 13 and was tagged by six markers, with the two top SNPs (S13_25716244 and S13_25667749) explaining the highest proportion of the total phenotypic variance (18%) and were separated by a distance of 0.05 Mb. Meanwhile, marker S13_25447531 (*p* = 8.56E-05) had the least proportion (16%) of the phenotypic variation. The second peak was tagged by marker S6_319697 (*p* = 6.31E-05) on chromosome 6. This marker explained 16% of the phenotypic variance for the trait (Table 2).

**Table 2:** Summary statistics of top significant SNPs at each major trait linked locus for root mealiness and other textural attributes of boiled cassava roots.

Trait	SNP_ID	Chr^a^	Position	Allele		MAF^b^	Value of *p*	*R* ^2^ ^c^
				Major	Minor			
Mealiness	S13_25716244	13	25,716,244	C	G	0.50	1.65E-05	0.18
Mealiness	S13_25667749	13	25,667,749	C	T	0.49	2.02E-05	0.18
Mealiness	S13_25591824	13	25,591,824	T	C	0.49	2.81E-05	0.17
Mealiness	S13_25417306	13	25,417,306	C	A	0.48	3.08E-05	0.17
Mealiness	S13_25394035	13	25,394,035	C	T	0.47	5.01E-05	0.17
Mealiness	S6_319697	6	319,697	T	A	0.06	6.31E-05	0.16
Mealiness	S13_25447531	13	25,447,531	A	C	0.48	8.56E-05	0.16
ADH^d^	S18_3604145	18	3604145	G	C	0.14	1.41E-06	0.21
ADH	S18_3573999	18	3,573,999	A	C	0.13	2.46E-06	0.20
ADH	S18_3563031	18	3,563,031	T	G	0.15	5.27E-06	0.18
ADH	S18_3414606	18	3,414,606	C	G	0.13	7.44E-06	0.18
ADH	S18_3414613	18	3,414,613	C	G	0.13	7.44E-06	0.18
ADH	S18_3297837	18	3,297,837	T	G	0.19	8.06E-06	0.18
Aroma	S17_7460784	17	7,460,784	C	T	0.11	9.90E-05	0.12
Colour	S1_25733720	1	25,733,720	T	C	0.44	9.05E-05	0.18
Colour	S1_25695204	1	25,695,204	A	G	0.49	9.85E-05	0.18
Colour	S1_25695218	1	25,695,218	G	T	0.49	9.85E-05	0.18
Fibre	S5_21198714	5	21,198,714	C	T	0.08	9.84E-05	0.16
Firmness	S4_23643811	4	23,643,811	A	G	0.43	1.08E-05	0.17
Taste	S10_3499386	10	3,499,386	A	G	0.24	5.02E-05	0.15
Taste	S1_24825469	1	24,825,469	C	A	0.49	5.32E-05	0.15
Taste	S10_3326655	10	3,326,655	A	T	0.24	6.42E-05	0.14
Taste	S10_3444639	10	3,444,639	G	T	0.24	7.98E-05	0.14
Taste	S10_3290580	10	3,290,580	C	T	0.27	9.07E-05	0.14

a *Chromosome*;

b *Minor allele frequency*;

c *Proportion of phenotypic variation explained by SNPs*;

d *Adhesiveness*.

#### Fibre

**Figure 5:**
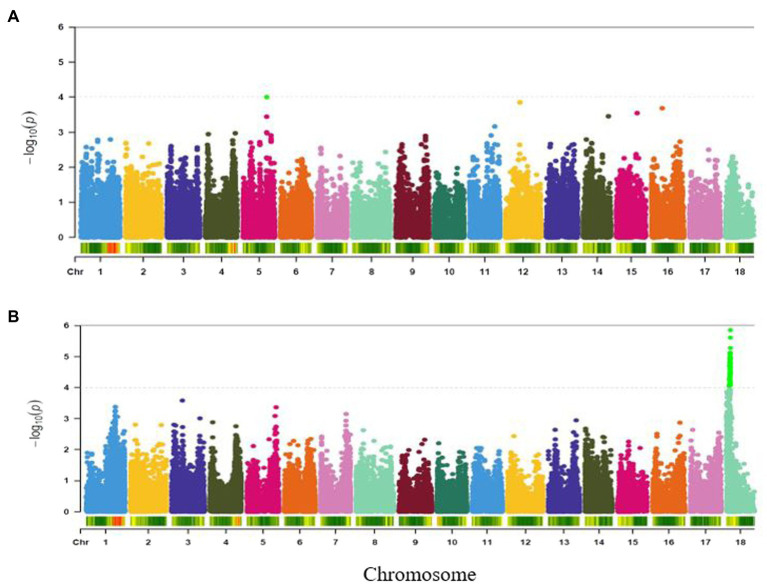
Manhattan plots for GWAS based on best linear unbiased predictions for **(A)** fibre, and **(B)** adhesiveness. The dashed horizontal line depicts the genome-wide significance threshold.

A single genomic region around 21.2 Mbp of chromosome 5 was found to be significantly associated with fibre (Figure 5A) and was tagged by SNP S5_21198714 (*p* = 9.84E-05). This marker explained 16% of the phenotypic variance for the trait (Table 2).

#### Adhesiveness

A total of 62 markers were significant for variation in adhesiveness. The significant SNPs associated with the trait were found in a single genomic region on chromosome 18 (around 32–35 Mb; Figure 5B). The peak SNP at this locus (S18_3604145, *p* = 1.41E-06) explained 21% of the trait variation (Table 2). Variance explained by each significant marker *R*^2^ varied from 14 to 21% (Supplementary Table S3).

#### Root Taste

**Figure 6:**
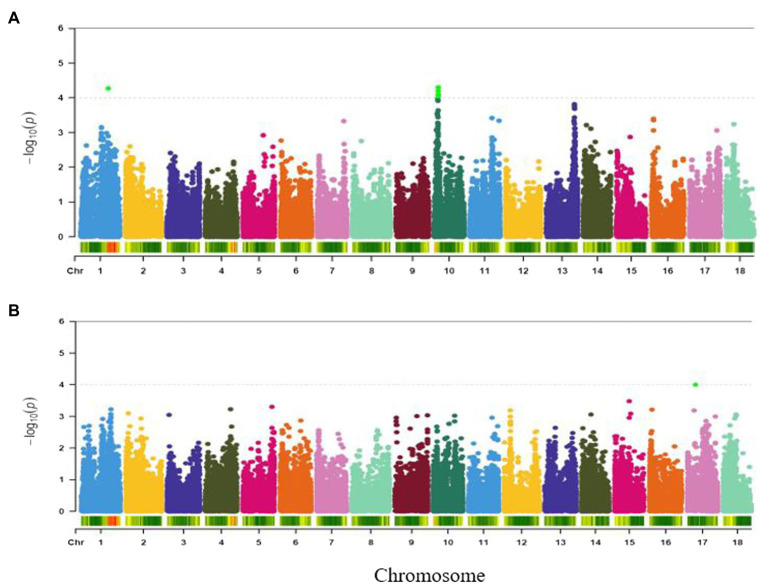
Manhattan plots for GWAS based on best linear unbiased predictions for **(A)** root taste, and **(B)** aroma. The dashed horizontal line depicts the genome-wide significance threshold.

Variation in taste of boiled roots was found to be associated with two major loci occurring on chromosomes 1 and 10 (Figure 6A). The most significant marker was S10_3499386 (*p* = 5.02E-05) followed by the marker on chromosome 1 (S1_24825469, *p* = 5.32E-05). These two SNPs accounted for the highest phenotypic variance (15%) for root taste (Table 2).

#### Aroma

A single genomic region on chromosome 17 (7.46 Mbp) was found to be linked to aroma (Figure 6B) and tagged by SNP S17_7460784 (*p* = 9.90E-05). The marker explained 12% of the trait variation (Table 2).

#### Colour

**Figure 7:**
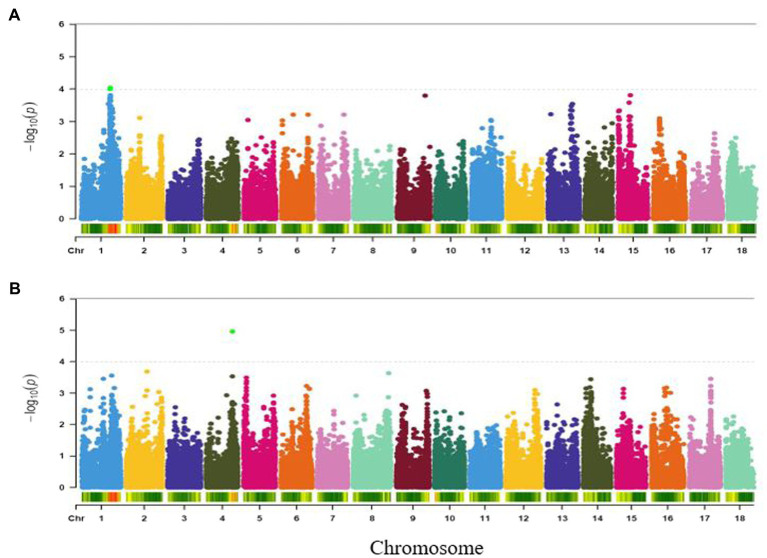
Manhattan plots for GWAS based on best linear unbiased predictions for **(A)** colour, and **(B)** firmness. The dashed horizontal line depicts the genome-wide significance threshold.

Three SNPs (S1_25733720, S1_25695204 and S1_25695218) with genome-wide association significance for colour variation in boiled roots were identified to be located within a single major locus on chromosome 1 (around 25.73 and 25.69 Mb regions; Figure 7A). Each of these markers explained 18% of the phenotypic variance for the trait (Table 2).

#### Firmness

Association analysis of firmness identified a significant locus around 23.64 Mbp region of chromosome 4 (Figure 7B) and tagged by marker S4_23643811 (*p* = 1.08E-05). The SNP marker explained 17% of the total phenotypic variation (Table 2).

### Putative Candidate Genes

The genomic regions encompassed by the top significant SNPs were further examined to identify annotated genes with putative functions using the *M.esculenta*_305_v6.1.gene.gff3 file of the cassava reference genome available on phytozome v12.1 (Goodstein et al., 2012). Candidate genes that are co-located with peak SNPs were identified and are presented in Table 3. The candidate gene Manes.13G026900, annotated as plant invertase/pectin methylesterase inhibitor superfamily protein, was found close to the top SNP S13_25716244 on chromosome 13, which was associated with natural variation in mealiness of boiled cassava roots. For fibre, a candidate gene Manes.05G153500, which encodes a walls-are-thin-1 (WAT1) protein, harboured the significant SNP S5_21198714 on chromosome 5. Two candidate genes, designated as Manes.18G037100 and Manes.18G037200 were found close to the top SNPs on chromosome 18 linked to adhesiveness. These genes encode for alpha-amylase. There were five potential candidate genes found in genomic regions associated with taste of boiled cassava roots on chromosome 1 and 10. These genes included Manes.01G123800 (encoding sucrose synthase), Manes.01G141200 (identified as beta-galactosidase-3), Manes.01G145700 (annotated as chalcone-flavanone-isomerase family protein), Manes.10G029400, which encodes for expansin-A4 protein, and Manes.10G034200 (encoding UDP-glucosyl-transferase-73B3). No known candidate genes were found in the vicinity of the significant marker S17_7460784 linked to aroma on chromosome 17. For colour of boiled cassava roots, two potential candidate genes Manes.01G141200 and Manes.01G147500 were co-located with the significant SNPs on chromosome1. The putative gene Manes.01G141200 encodes for beta-galactosidase-3 while the Manes.01G147500 putative gene encodes a Myb-like domain protein. Also, the significant SNPs associated with colour of boiled cassava roots were furthest downstream from the phytoene-synthase (PSY) gene Manes.01G124200, which is known to increase the accumulation of total carotenoid content (TCC) in cassava roots. Eight candidate genes near the significant SNP S4_23643811 on chromosome 4 linked to firmness encode for a wall-associated kinase family protein (WAKs). These genes included Manes.04G099400, Manes.04G099500, Manes.04G099900, Manes.04G100100, Manes.04G100200, Manes.04G100300, Manes.04G100400, and Manes.04G100500.

**Table 3:** Candidate genes co-located with significant SNPs associated with mealiness and other textural attributes of boiled cassava roots.

Trait	Chr^a^	Position	Candidate gene	Gene annotation
Mealiness	S13	25,716,244	Manes.13G026900	Plant invertase/pectin methylesterase inhibitor
Fibre	S5	21,198,714	Manes.05G153500	Walls-Are-Thin-1 protein (WAT1)
Adhesiveness	S18	3,604,145	Manes.18G037100, Manes.18G037200	Alpha-amylase
Taste	S1	24,825,469	Manes.01G123800	Sucrose synthase
			Manes.01G141200	Beta-galactosidase-3
			Manes.01G145700	Chalcone-flavanone isomerase family protein
	S10	3,499,386	Manes.10G029400	Expansin-A4 protein
			Manes.10G034200	UDP-glucosyl-transferase-73B3
Aroma	S17	7,460,784	None	–
Colour	S1	25,733,720	Manes.01G141200, Manes.01G147500	Beta-galactosidase-3, Myb-like domain protein
		25,695,204	Manes.01G141200, Manes.01G147500	Beta-galactosidase-3, Myb-like domain protein
		25,695,218	Manes.01G141200, Manes.01G147500	Beta-galactosidase-3, Myb-like domain protein
Firmness	S4	23,643,811	Manes.04G099400, Manes.04G099500, Manes.04G099900, Manes.04G100100, Manes.04G100200, Manes.04G100300, Manes.04G100400, Manes.04G100500	Wall-associated kinase family protein

a *Chromosome*.

## Discussion

The texture of boiled cassava roots is a major determinant of consumer acceptance and preference. Although several studies have been conducted on the quality attributes of boiled cassava roots, the genetic architecture underlying these textural attributes remains unexplored. This study aimed to identify QTL regions and potential candidate genes associated with genetic variation in root mealiness and other quality attributes of boiled cassava roots using a collection of 142 accessions from the NRCRI cassava breeding programme in Nigeria.

A total of 59,792 SNPs were found to be distributed across the 18 chromosomes at various densities, in which 80 SNPs were determined to associate with root mealiness and other textural attributes of boiled cassava roots. The average PIC value of 0.26 obtained in the present study is higher than that reported by Udoh et al. (2017) but comparable to those previously reported in other studies (Simko et al., 2012; Zhang et al., 2016; Adewale et al., 2020). This shows the informativeness of the SNP markers used in this study. Estimation of population structure and individual relatedness in association studies is required to minimize spurious associations (Yu et al., 2006). The 142 white and yellow-root accessions used in this study were classified into four subgroups. For all the subgroups, both white and yellow accessions were distributed, indicating that these samples are suitable for association analysis. The three complementary approaches employed in this study, namely DAPC, NJ phylogenetic tree, and admixture ancestry to define the optimal number of subgroups all revealed the presence of four genetic subgroups among the 142 cassava accessions. Similar conclusion about the number of distinct groups has been drawn in cassava (do Carmo et al., 2020). The LD decay over physical distance is crucial in determining the number and density of markers required for genomic analyses such as GWAS (Flint-Garcia et al., 2003; do Carmo et al., 2020). We observed LD decay in the population composed of white and yellow accessions at approximately 30 kb, which is higher than that in Brazilian cassava germplasm (~20 kb; Albuquerque et al., 2018) and elite IITA cassava breeding lines (10 kb; Rabbi et al., 2020), but lower compared to East–West African breeding lines (~50 kb; Wolfe et al., 2016). The observed LD level in this study implies that higher-resolution map would be achieved when using this population.

Genome-wide association study has been widely used in deciphering the genetic basis of complex traits in various crop plants (Huang et al., 2010; de Oliveira et al., 2012; Morris et al., 2013; Sukumaran et al., 2018); nonetheless, complex genetic architectures and structured characters can produce false signals and indirect relationships in GWAS. The model fitness for the association analysis was based on the quantile–quantile (Q-Q) plot, which is a plot of observed versus expected values of *p* under the null hypothesis that there is no relationship between a SNP marker and the phenotype. The results showed that the majority of the points in the Q-Q plots were aligned on the diagonal line for all the traits studied, implying that false allelic associations due to population structure and relative kinship were largely minimized. This finding supports earlier reports by Kuki et al. (2018) and Adewale et al. (2020).

The discovery of QTL and genes influencing the textural attributes of boiled cassava roots is of great importance to marker-assisted breeding. Results from the GWAS analysis provide the first insights into the genetic architecture of boiled cassava root texture. In the present study, GWAS identified 80 significant SNP markers associated with mealiness, fibre, adhesiveness, taste, aroma, colour and firmness at the threshold of −log(P) = 4. These markers were found on chromosomes 1, 4, 5, 6, 10, 13, 17, and 18. The significant markers identified using GWAS, particularly those co-located with candidate genes for the studied traits can provide an important reference for the development functional markers. These gene-based functional markers would be potentially useful in MAS to improve boiled cassava root texture.

The most notable candidate gene identified in this study is found in the vicinity occupied by SNP S13_25716244 on chromosome 13. SNP S13_25716244 is co-located with pectin-methylesterase inhibitor (PMEI) gene (Manes.13G026900), a proteinaceous inhibitor of pectin-methylesterase (PME). The protein PMEI has been reported to be present in the aqueous phase of fully ripe fruit, and is probably synthesized as a larger precursor, undergoing proteolytic cleavage during the fruit ripening process (Giovane et al., 1995). It has been shown that the action of PME on pectin affects the food textural quality of plant-based food products, either favourably or deleteriously, depending on the product at hand (Jolie et al., 2010). This finding is particularly interesting as it is well known that pectin is recognized as being responsible for cell adhesion and for the mealiness or crispness of vegetables and fruits (Parker et al., 2001; Jarvis et al., 2003). Some evidence of PME activity in cooked potato texture have been reported by various studies (Ross et al., 2010, 2011a,b; Lloyd, 2011).

On chromosome 5, the SNP S5_21198714 was associated with fibre. This SNP was found close to the gene Manes.05G153500, which encodes a walls-are-thin 1 (WAT1) protein. WAT1 gene is a member protein that belongs to the plant drug/metabolite exporter (P-DME) family (Jack et al., 2001) and has been reported to have a regulation role in secondary cell wall in fibres of Arabidopsis (Ranocha et al., 2010). The secondary cell wall can be regarded as a complex, natural composite substance and consists of three major components in angiosperms. These components included cellulose, xylan, and lignin. Ranocha et al. (2010) reported that the WAT1 phenotype can be associated with the transcriptional downregulation of genes known to play a pivotal role in the biosynthesis of secondary cellulose, xylan and lignin in WAT1-1 stems and metabolites in lignin biosynthesis. The authors found WAT1 gene to be ubiquitously expressed all over the plant with its preferential expression associated with vascular tissues, including developing xylem vessels and fibres. BLAST searches have shown that WAT1 gene has homologs in different plant species, including rice (*Oryza sativa*), poplar (*Populus trichocarpa* × *Populus deltoides*), maize (*Zea mays*) and the moss, *Physcomitrella patens* (Ranocha et al., 2010). The SNP marker S5_21198714 indicated that the candidate gene (WAT1) identified in this study could be associated with fibre development or secondary cell wall formation in cassava. The gene therefore could be a promising target to further decipher the genetic architecture of boiled cassava root texture and should be further tested in cassava breeding programmes to determine its usefulness in selecting genotypes with good culinary qualities.

Two putative genes, Manes.18G037100 and Manes.18G037200, within location of the SNPs associated with adhesiveness on chromosome 18 encode for alpha-amylase. The alpha-amylase gene performs important functions and roles in carbohydrate metabolism. The importance of this protein in initiating starch degradation has been reported in different starchy crops, including cassava (Oyewole and Odunfa, 1992; do Carmo et al., 2020). Lengbamroung et al. (2005) found a high expression of the gene encoding alpha-amylase protein in cassava storage roots. In cassava, the genes for alpha-amylase and beta-amylase were co-located with significant polymorphisms identified for waxy starch (do Carmo et al., 2020). Thus, the alpha-amylase gene may be a waxy gene (*Wx*) involved in the expression of waxy phenotype in cassava. Yang et al. (2018b) reported the effects of the *Wx* gene on rice eating and cooking qualities. It was also reported that two enzymes, PME and amylase, played an important role in firmness in sweet potato (He et al., 2014). In this study, the alpha-amylase gene was located on chromosome 18, where significant SNPs associated with adhesiveness were identified. This possibly suggests that the gene might have a critical influence on boiled cassava root texture. Nonetheless, further investigations will be needed to fully understand the contribution of the alpha-amylase gene to boiled cassava root texture.

The candidate gene (Manes.01G141200) encoding beta-galactosidase (β-Gal) was found around the genomic regions of the significant SNPs associated with variation in taste and colour of boiled cassava roots on chromosome 1. Identifying this potential candidate gene influencing these two traits on chromosome 1 QTL region strongly indicates the presence of pleiotropic effects. β-Gal, one of the major cell wall modifying enzymes (a pectin enzyme), plays an important role in fruit ripening and softening (Yang et al., 2018a). It is known to facilitate fruit ripening and softening by increasing the cell wall porosity as well as enhancing the access of other cell wall degrading enzymes (Goulao et al., 2007; Ng et al., 2015). It has been shown that alterations in the β-Gal activity resulted in differences in pectin solubilization and the cell wall structures, and among apple varieties with different textures during fruit ripening (Yang et al., 2018a). β-Gals are basically encoded by members of large gene families and their roles have been investigated in many plant species, including arabidopsis (Ahn et al., 2007), strawberry (Trainotti et al., 2001), tomato (Smith and Gross, 2000), pear (Tateishi et al., 2001), and papaya (do Prado et al., 2016). In addition to β-Gal, the following candidate genes were found in the genomic regions associated with variation in taste of boiled cassava roots on chromosomes 1 and 10: Manes.01G123800 (encoding sucrose synthase), Manes.01G145700 (identified as chalcone-flavanone isomerase family protein), Manes.10G029400 (annotated as expansin-A4 protein), and Manes.10G034200 (encoding UDP-glucosyltransferase-73B3). These genes have also been previously reported to play important roles in fruit ripening and softening, and flavonoid biosynthesis (Jiang et al., 2015; Minoia et al., 2016; Song et al., 2016; Zhao et al., 2017). However, these putative genes have not been well studied in cassava and need further investigations to better understand their potential roles in cassava, particularly related to textural attributes of boiled cassava roots.

A potential candidate gene (Manes.01G147500) encoding Myb-like domain protein was found closer to the significant SNPs identified for colour on chromosome 1. Compared to animals, plants contain a Myb-protein subfamily that is characterized by the R2R3-type Myb domain (Stracke et al., 2001). Previous genetic studies have revealed that the yellow seed1 (Y1) gene, which controls pericarp pigmentation and phytoalexin production in sorghum, encodes a R2R3-type of Myb domain protein that controls the expression of chalcone synthase, chalcone isomerase, and dihydroflavonol reductase genes required for 3-deoxyflavonoids biosynthesis (Boddu et al., 2005, 2006; Ibraheem et al., 2010; Morris et al., 2013). In plants, Myb genes are believed to play an important role in regulating pigment biosynthesis pathway (Nesi et al., 2001; Kobayashi et al., 2002; Himi and Noda, 2005; Allan et al., 2008). This has been demonstrated in wheat as well as in maize (Grotewold et al., 1994; Dong et al., 2001; Himi et al., 2002; Lee and Harper, 2002; Chopra et al., 2003; Boddu et al., 2006). In cassava, the Myb gene was found within the location occupied by association hit for petiole colour and leaf greenness on chromosome 1 (Rabbi et al., 2020); however, it will be necessary to further understand the contribution of the Myb gene in the epigenetic regulation of the anthocyanin biosynthesis pathway in cassava. Moreover, the significant polymorphisms associated with colour of boiled roots were located downstream of the phytoene synthase gene (Manes.01G124200). The identified candidate gene is responsible for the increased accumulation of provitamin A carotenoid in cassava roots (Welsch et al., 2010). This finding corroborates earlier GWAS reports on TCC in cassava (Esuma et al., 2016; Rabbi et al., 2017, 2020; Ikeogu et al., 2019).

Eight candidate genes encoding wall-associated kinase family protein were found to be located close to the significant SNP S4_23643811 associated with variation in firmness of boiled cassava roots on chromosome 4. Wall-associated kinases (WAKs) are known for their developmental role in cell expansion (Decreux and Messiaen, 2005; Kohorn, 2016). It has been shown that pectinase, an enzyme responsible for degrading pectin present in the cell wall releases WAKs (Kohorn and Kohorn, 2012). This finding is interesting as it has been reported that pectin degradation may affect the fruit firmness of apple cultivars (Yang et al., 2018a). WAK-like genes have been identified in different plant species including arabidopsis, tomato, rice as well as in maize (Zhang et al., 2005; Rosli et al., 2013; Hurni et al., 2015; Zuo et al., 2015). Nonetheless, this potential gene has not been studied in cassava and needs further exploration to better understand its relationship with pectin and their impact on the texture of boiled cassava roots.

The findings presented in this study have increased our understanding of the underlying genetic basis of boiled cassava root texture. Through GWAS, we identified novel genes including a promising gene for root mealiness, which subsequently on validation, could be potential targets for breeders in marker-assisted breeding to improve cassava culinary qualities.

## Conclusion

The present study describes the first genome-wide association mapping approach in cassava to decipher the genetic architecture of root mealiness and other important quality attributes of boiled cassava roots. The study identified 80 significant SNPs associated with variation in measured traits. These polymorphisms can be further explored for use in MAS of the studied traits to increase their selection efficiency and the rate of genetic gain. This study also identified relevant candidate genes for the traits studied except for aroma. The novel gene Manes.13G026900 on chromosome 13 could be invaluable for the development of cassava genotypes with desirable mealiness values through marker-assisted breeding and genomic selection. In addition, chromosome 1 encompassed genomic regions harbouring promising genes for taste, colour and other texture-related traits of boiled cassava roots. Further genetic studies involving transcript/transcriptome analysis, fine mapping, joint linkage mapping and mapping using different mapping populations will be required to validate associations and candidate genes identified in this study so that marker-assisted breeding approaches could be used as ideal tools to accelerate genetic improvement of root mealiness and other textural traits in African cassava germplasm, particularly in Nigeria where cassava is a major staple food.

## Data Availability Statement

The datasets presented in this study can be found in online repositories. The names of the repository/repositories and accession number(s) can be found in the article/Supplementary Material.

## Author Contributions

KU and CE conceived, designed and conducted the study. DN provided the plant material and high-quality SNPs used in the study. KU and AP analyzed the data. IR assisted KU in candidate gene analysis. DD, PT, SO, and CE supervised KU. KU generated the phenotypic data, interpreted the results and wrote the manuscript. DN, IR, DD, PT, SO, and CE revised the manuscript. All authors have read and approved the final version of the manuscript.

## Funding

This study was supported by the Deutscher Akademischer Austauschdienst (DAAD) to pursue a Ph.D in Plant Breeding at the West Africa Centre for Crop Improvement (WACCI), University of Ghana, Legon, Accra, Ghana. We acknowledge support from Next Generation Cassava Breeding Project through funds received from the Bill and Melinda Gates Foundation (BMGF) and the Department for International Development of the United Kingdom (UKAid; Grant number OPP1048542, http://www.gatesfoundation.org).

## Conflict of Interest

The authors declare that the research was conducted in the absence of any commercial or financial relationships that could be construed as a potential conflict of interest.

## Publisher’s Note

All claims expressed in this article are solely those of the authors and do not necessarily represent those of their affiliated organizations, or those of the publisher, the editors and the reviewers. Any product that may be evaluated in this article, or claim that may be made by its manufacturer, is not guaranteed or endorsed by the publisher.

## Footnotes

^1^ http://phytozome.jgi.doe.gov

^2^ http://go.pantherdb.org/

## References

[ref1] Adewale S. A. Badu-Apraku B. Akinwale R. O. Paterne A. A. Gedil M. Garcia-Oliveira A. L. (2020). Genome-wide association study of striga resistance in early maturing white tropical maize inbred lines. BMC Plant Biol. 20:203. doi: 10.1186/s12870-020-02360-0, PMID: 32393176 PMC7212567

[ref2] Ahn Y. O. Zheng M. Bevan D. R. Esen A. Shiu S. H. Benson J.. (2007). Functional genomic analysis of *Arabidopsis thaliana* glycoside hydrolase family 35. Phytochemistry 68, 1510–1520. doi: 10.1016/j.phytochem.2007.03.021, PMID: 17466346

[ref3] Albuquerque H. Y. G. Carmo C. D. Brito A. C. Oliveira E. J. (2018). Genetic diversity of *Manihot esculenta* Crantz germplasm based on single-nucleotide polymorphism markers. Ann. Appl. Biol. 173, 271–284. doi: 10.1111/aab.12460, PMID: 34314448

[ref4] Alexander D. H. Novembre J. Lange K. (2009). Fast model-based estimation of ancestry in unrelated individuals. Genome Res. 19, 1655–1664. doi: 10.1101/gr.094052.109, PMID: 19648217 PMC2752134

[ref5] Allan A. C. Hellens R. P. Laing W. A. (2008). MYB transcription factors that colour our fruit. Trends Plant Sci. 13, 99–102. doi: 10.1016/j.tplants.2007.11.012, PMID: 18280199

[ref6] Batra S. Carlson J. Hayes R. Shu S. Schmutz J. Rokhsar D. (2014). Phytozome Comparative Plant Genomics Portal (Berkeley, CA, USA: Lawrence Berkeley National Lab), 1–2.

[ref7] Boddu J. Jiang C. Sangar V. Olson T. Peterson T. Chopra S. (2006). Comparative structural and functional characterization of sorghum and maize duplications containing orthologous Myb transcription regulators of 3-deoxyflavonoid biosynthesis. Plant Mol. Biol. 60, 185–199. doi: 10.1007/s11103-005-3568-1, PMID: 16429259

[ref8] Boddu J. Svabek C. Ibraheem F. Jones A. D. Chopra S. (2005). Characterization of a deletion allele of a sorghum Myb gene yellow seedl showing loss of 3-deoxyflavonoids. Plant Sci. 169, 542–552. doi: 10.1016/j.plantsci.2005.05.007

[ref9] Ceballos H. Kawuki R. S. Gracen V. E. Yencho G. C. Hershey C. H. (2015). Conventional breeding, marker-assisted selection, genomic selection and inbreeding in clonally propagated crops: a case study for cassava. Theor. Appl. Genet. 128, 1647–1667. doi: 10.1007/s00122-015-2555-4, PMID: 26093610 PMC4540783

[ref10] Chang C. C. Chow C. C. Tellier L. C. Vattikuti S. Purcell S. M. Lee J. J. (2015). Second-generation PLINK: rising to the challenge of larger and richer datasets. GigaScience 4:7. doi: 10.1186/s13742-015-0047-8, PMID: 25722852 PMC4342193

[ref11] Chopra S. Cocciolone S. M. Bushman S. Sangar V. McMullen M. D. Peterson T. (2003). The maize unstable factor for orange1 is a dominant epigenetic modifier of a tissue specifically silent allele of pericarp color1. Genetics 163, 1135–1146. doi: 10.1093/genetics/163.3.1135, PMID: 12663550 PMC1462483

[ref12] Danecek P. (2011). 1000 genomes project analysis group. The variant call format and VCFtools. Bioinformatics 27, 2156–2158. doi: 10.1093/bioinformatics/btr330, PMID: 21653522 PMC3137218

[ref13] de Oliveira E. J. de Resende M. D. V. da Silva Santos V. Ferreira C. F. Oliveira G. A. F. da Silva M. S.. (2012). Genome-wide selection in cassava. Euphytica 187, 263–276. doi: 10.1007/s10681-012-0722-0, PMID: 34784936

[ref14] Decreux A. Messiaen J. (2005). Wall-associated kinase WAK1 interacts with cell wall pectins in a calcium-induced conformation. Plant Cell Physiol. 46, 268–278. doi: 10.1093/pcp/pci026, PMID: 15769808

[ref15] do Carmo C. D. e Sousa M. B. Brito A. C. de Oliveira E. J. (2020). Genome-wide association studies for waxy starch in cassava. Euphytica 216:82. doi: 10.1007/s10681-020-02615-9

[ref16] do Prado S. B. R. Melfi P. R. Castro-Alves V. C. Broetto S. G. Araujo E. S. do Nascimento J. R. O.. (2016). Physiological degradation of pectin in papaya cell walls: release of long chains galacturonans derived from insoluble fractions during postharvest fruit ripening. Front. Plant Sci. 7:1120. doi: 10.3389/fpls.2016.01120, PMID: 27512402 PMC4961711

[ref17] Dong X. Braun E. L. Grotewold E. (2001). Functional conservation of plant secondary metabolic enzymes revealed by complementation of Arabidopsis flavonoid mutants with maize genes. Plant Physiol. 127, 46–57. doi: 10.1104/pp.127.1.46, PMID: 11553733 PMC117961

[ref18] Elshire R. J. Glaubitz J. C. Sun Q. Poland J. A. Kawamoto K. Buckler E. S.. (2011). A robust, simple genotyping-by-sequencing (GBS) approach for high diversity species. PLoS One 6:e19379. doi: 10.1371/journal.pone.0019379, PMID: 21573248 PMC3087801

[ref19] Esuma W. Herselman L. Labuschagne M. T. Ramu P. Lu F. Baguma Y.. (2016). Genome-wide association mapping of provitamin A carotenoid content in cassava. Euphytica 212, 97–110. doi: 10.1007/s10681-016-1772-5, PMID: 29293815

[ref20] Ezenwaka L. C. Del Carpio D. P. Jannink J. L. Rabbi I. Y. Danquah E. Asante I.. (2018). Genome-wide association study of resistance to cassava green mite pest and related traits in cassava. Crop Sci. 58, 1907–1918. doi: 10.2135/cropsci2018.01.0024, PMID: 32734418

[ref21] Favaro S. P. Beleia A. da Silva Fonseca N. Jr. Waldron K. W. (2008). The roles of cell wall polymers and intracellular components in the thermal softening of cassava roots. Food Chem. 108, 220–227. doi: 10.1016/j.foodchem.2007.10.070

[ref22] Flint-Garcia S. A. Thornsberry J. M. Buckler E. S. (2003). Structure of linkage disequilibrium in plants. Annu. Rev. Plant Biol. 54, 357–374. doi: 10.1146/annurev.arplant.54.031902.134907, PMID: 14502995

[ref23] Gao L. Turner M. K. Chao S. Kolmer J. Anderson J. A. (2016). Genome wide association study of seedling and adult plant leaf rust resistance in elite spring wheat breeding lines. PLoS One 11:e0148671. doi: 10.1371/journal.pone.0148671, PMID: 26849364 PMC4744023

[ref24] Giovane A. Balestrieri C. Quagliuolo L. Castaldo D. Servillo L. (1995). A glycoprotein inhibitor of pectin methylesterase in kiwi fruit. Purification by affinity chromatography and evidence of a ripening-related precursor. Eur. J. Biochem. 233, 926–929. doi: 10.1111/j.1432-1033.1995.926_3.x, PMID: 8521860

[ref25] Glaubitz J. C. Casstevens T. M. Lu F. Harriman J. Elshire R. J. Sun Q.. (2014). TASSEL-GBS: a high capacity genotyping by sequencing analysis pipeline. PLoS One 9:e90346. doi: 10.1371/journal.pone.0090346, PMID: 24587335 PMC3938676

[ref26] Goodstein D. M. Shu S. Howson R. Neupane R. Hayes R. D. Fazo J.. (2012). Phytozome: a comparative platform for green plant genomics. Nucleic Acids Res. 40, D1178–D1186. doi: 10.1093/nar/gkr944, PMID: 22110026 PMC3245001

[ref27] Goulao L. F. Santos J. de Sousa I. Oliveira C. M. (2007). Patterns of enzymatic activity of cell wall-modifying enzymes during growth and ripening of apples. Postharvest Biol. Technol. 43, 307–318. doi: 10.1016/j.postharvbio.2006.10.002

[ref28] Grotewold E. Drummond B. J. Bowen B. Peterson T. (1994). The Myb-homologous P gene controls phlobaphene pigmentation in maize floral organs by directly activating a flavonoid biosynthetic gene subset. Cell 76, 543–554. doi: 10.1016/0092-8674(94)90117-1, PMID: 8313474

[ref29] Hamblin M. T. Rabbi I. Y. (2014). The effects of restriction-enzyme choice on properties of genotyping-by-sequencing libraries: a study in cassava (*Manihot esculenta* Crantz). Crop Sci. 54, 2603–2608. doi: 10.2135/cropsci2014.02.0160

[ref30] He J. Cheng L. Gu Z. Hong Y. Li Z. (2014). Effects of low-temperature blanching on tissue firmness and cell wall strengthening during sweetpotato flour processing. Int. J. Food Sci. Technol. 49, 1360–1366. doi: 10.1111/ijfs.12437

[ref31] Himi E. Mares D. J. Yanagisawa A. Noda K. (2002). Effect of grain colour gene (R) on grain dormancy and sensitivity of the embryo to abscisic acid (ABA) in wheat. J. Exp. Bot. 53, 1569–1574. doi: 10.1093/jxb/erf005, PMID: 12096095

[ref32] Himi E. Noda K. (2005). Red grain colour gene (R) of wheat is a Myb-type transcription factor. Euphytica 143, 239–242. doi: 10.1007/s10681-005-7854-4, PMID: 14718498

[ref33] Hongbété F. Mestres C. Akissoé N. Pons B. Hounhouigan J. D. Cornet D.. (2011). Effects of cultivar and harvesting conditions (age, season) on the texture and taste of boiled cassava roots. Food Chem. 126, 127–133. doi: 10.1016/j.foodchem.2010.10.088

[ref34] Huang X. Wei X. Sang T. Zhao Q. Feng Q. Zhao Y.. (2010). Genome-wide association studies of 14 agronomic traits in rice landraces. Nat. Genet. 42, 961–967. doi: 10.1038/ng.695, PMID: 20972439

[ref35] Hurni S. Scheuermann D. Krattinger S. G. Kessel B. Wicker T. Herren G.. (2015). The maize disease resistance gene Htn1 against northern corn leaf blight encodes a wall-associated receptor-like kinase. Proc. Natl. Acad. Sci. U. S. A. 112, 8780–8785. doi: 10.1073/pnas.1502522112, PMID: 26124097 PMC4507197

[ref36] Ibraheem F. Gaffoor I. Chopra S. (2010). Flavonoid phytoalexin-dependent resistance to anthracnose leaf blight requires a functional yellow seed1 in *Sorghum bicolor*. Genetics 184, 915–926. doi: 10.1534/genetics.109.111831, PMID: 20083611 PMC2865927

[ref37] ICGMC (2015). High-resolution linkage map and chromosome-scale genome assembly for cassava (*Manihot esculenta* Crantz) from 10 populations. G3 5, 133–144. doi: 10.1534/g3.114.015008, PMID: 25504737 PMC4291464

[ref38] Ikeogu U. N. Akdemir D. Wolfe M. D. Okeke U. G. Chinedozi A. Jannink J.-L.. (2019). Genetic correlation, genome-wide association and genomic prediction of portable NIRS predicted carotenoids in cassava roots. Front. Plant Sci. 10:1570. doi: 10.3389/fpls.2019.01570, PMID: 31867030 PMC6904298

[ref39] Iragaba P. Nuwamanya E. Wembabazi E. Baguma Y. Dufour D. Earle E. D.. (2019). Estimates for heritability and consumer-validation of a penetrometer method for phenotyping softness of cooked cassava roots. Afr. Crop. Sci. J. 27, 147–163. doi: 10.4314/acsj.v27i2.3

[ref40] Jack D. L. Yang N. M. Saier M. H. Jr. (2001). The drug/metabolite transporter superfamily. Eur. J. Biochem. 268, 3620–3639. doi: 10.1046/j.1432-1327.2001.02265.x, PMID: 11432728

[ref41] Jarvis M. C. Briggs S. P. H. Knox J. P. (2003). Intercellular adhesion and cell separation in plants. Plant Cell Environ. 26, 977–989. doi: 10.1046/j.1365-3040.2003.01034.x, PMID: 33955495

[ref42] Jiang W. Yin Q. Wu R. Zheng G. Liu J. Dixon R. A.. (2015). Role of a chalcone isomerase-like protein in flavonoid biosynthesis in arabidopsis *thaliana*. J. Exp. Bot. 66, 7165–7179. doi: 10.1093/jxb/erv413, PMID: 26347569 PMC4765788

[ref43] Jiwuba L. Danquah A. Asante I. Blay E. Onyeka J. Danquah E.. (2020). Genotype by environment interaction on resistance to cassava green mite associated traits and effects on yield performance of cassava genotypes in Nigeria. Front. Plant Sci. 11:572200. doi: 10.3389/fpls.2020.572200, PMID: 33013995 PMC7498573

[ref44] Jolie R. P. Duvetter T. Van Loey A. M. Hendrickx M. E. (2010). Pectin methylesterase and its proteinaceous inhibitor: a review. Carbohydr. Res. 345, 2583–2595. doi: 10.1016/j.carres.2010.10.002, PMID: 21047623

[ref45] Jombart T. Devillard S. Balloux F. (2010). Discriminant analysis of principal components: a new method for the analysis of genetically structured populations. BMC Genet. 11:94. doi: 10.1186/1471-2156-11-94, PMID: 20950446 PMC2973851

[ref46] Kayondo S. I. Del Carpio D. P. Lozano R. Ozimati A. Wolfe M. D. Baguma Y.. (2018). Genome-wide association mapping and genomic prediction for CBSD resistance in *Manihot esculenta*. Sci. Rep. 8:1549. doi: 10.1038/s41598-018-19696-1, PMID: 29367617 PMC5784162

[ref47] Kobayashi S. Ishimaru M. Hiraoka K. Honda C. (2002). Myb-related genes of the Kyoho grape (*Vitis labruscana*) regulate anthocyanin biosynthesis. Planta 215, 924–933. doi: 10.1007/s00425-002-0830-5, PMID: 12355152

[ref48] Kohorn B. D. (2016). Cell wall-associated kinases and pectin perception. J. Exp. Bot. 67, 489–494. doi: 10.1093/jxb/erv467, PMID: 26507892

[ref49] Kohorn B. D. Kohorn S. L. (2012). The cell wall-associated kinases, WAKs, as pectin receptors. Front. Plant Sci. 3:88. doi: 10.3389/fpls.2012.00088, PMID: 22639672 PMC3355716

[ref50] Kuki M. C. Scapim C. A. Rossi E. S. Mangolin C. A. do Amaral A. T. Jr. Pinto R. J. B. (2018). Genome wide association study for gray leaf spot resistance in tropical maize core. PLoS One 13:e0199539. doi: 10.1371/journal.pone.0199539, PMID: 29953466 PMC6023161

[ref51] Lee E. A. Harper V. (2002). Suppressor of Pericarp Pigmentation 1 (SPP1), a novel gene involved in phlobaphene accumulation in maize (*Zea mays* L.) pericarps. Maydica 47, 51–58.

[ref52] Lengbamroung P. Vichukit V. Visser R. G. F. Nakasathien S. (2005). Cloning and molecular characterization of α- and β-amylase genes from Cassava (*Manihot esculenta* Crantz). Nat. Sci. 39, 446–454.

[ref53] LiLin-Yin (2019). CMplot: Circle Manhattan plot. Available at: https://github.com/YinLiLin/CMplot (Accessed: 19 July, 2020).

[ref54] Lipka A. E. Tian F. Wang Q. Peiffer J. Li M. Bradbury P. J.. (2012). GAPIT: genome association and prediction integrated tool. Bioinformatics 28, 2397–2399. doi: 10.1093/bioinformatics/bts444, PMID: 22796960

[ref55] Lloyd D. (2011). Genetic control of cooked potato tuber taste and texture. PhD thesis. University of Dundee.

[ref56] Minoia S. Boualem A. Marcel F. Troadec C. Quemener B. Cellini F.. (2016). Induced mutations in tomato SlExp1 alter cell wall metabolism and delay fruit softening. Plant Sci. 242, 195–202. doi: 10.1016/j.plantsci.2015.07.001, PMID: 26566837

[ref57] Misra G. Badoni S. Domingo C. J. Cuevas R. P. O. Llorente C. Mbanjo E. G. N.. (2018). Deciphering the genetic architecture of cooked rice texture. Front. Plant Sci. 9:1405. doi: 10.3389/fpls.2018.01405, PMID: 30333842 PMC6176215

[ref58] Mogga M. Sibiya J. Shimelis H. Lamo J. Yao N. (2018). Diversity analysis and genome-wide association studies of grain shape and eating quality traits in rice (*Oryza sativa* L.) using DArT markers. PLoS One 13:e0198012. doi: 10.1371/journal.pone.0198012, PMID: 29856872 PMC5983461

[ref59] Morris G. P. Ramu P. Deshpande S. P. Hash C. T. Shah T. Upadhyaya H. D.. (2013). Population genomic and genome-wide association studies of agroclimatic traits in sorghum. Proc. Natl. Acad. Sci. 110, 453–458. doi: 10.1073/pnas.1215985110, PMID: 23267105 PMC3545811

[ref60] Nesi N. Jond C. Debeaujon I. Caboche M. Lepiniec L. (2001). The arabidopsis TT2 gene encodes an R2R3 MYB domain protein that acts as a key determinant for proanthocyanidin accumulation in developing seed. Plant Cell 13, 2099–2114. doi: 10.1105/TPC.010098, PMID: 11549766 PMC139454

[ref61] Ng J. K. T. Schröder R. Brummell D. A. Sutherland P. W. Hallett I. C. Smith B. G.. (2015). Lower cell wall pectin solubilisation and galactose loss during early fruit development in apple (*Malus × domestica*) cultivar ‘Scifresh’ are associated with slower softening rate. J. Plant Physiol. 176, 129–137. doi: 10.1016/j.jplph.2014.12.012, PMID: 25602611

[ref62] Ngeve J. M. (2003). Cassava root yields and culinary qualities as affected by harvest age and test environment. J. Sci. Food Agric. 83, 249–257. doi: 10.1002/jsfa.1307

[ref63] Nweke F. I. Spencer D. S. C. Lynam J. K. (2002). The Cassava Transformation: Africa’s Best Kept Secret. East Lansing, Michigan, USA: Michigan State University Press.

[ref64] Oyewole O. B. Odunfa S. A. (1992). Extracellular enzyme activities during cassava fermentation for ‘fufu’ production. World J. Microbiol. Biotechnol. 8, 71–72. doi: 10.1007/BF01200690, PMID: 24425340

[ref65] Paradis E. Claude J. Strimmer K. (2004). APE: analyses of phylogenetics and evolution in R language. Bioinformatics 20, 289–290. doi: 10.1093/bioinformatics/btg412, PMID: 14734327

[ref66] Parker C. C. Parker M. L. Smith A. C. Waldron K. W. (2001). Pectin distribution at the surface of potato parenchyma cells in relation to cell-cell adhesion. J. Agric. Food Chem. 49, 4364–4371. doi: 10.1021/jf0104228, PMID: 11559139

[ref67] Prochnik S. Marri P. R. Desany B. Rabinowicz P. D. Kodira C. Mohiuddin M.. (2012). The cassava genome: current progress, future directions. Trop. Plant Biol. 5, 88–94. doi: 10.1007/s12042-011-9088-z, PMID: 22523606 PMC3322327

[ref68] Purcell S. Neale B. Todd-Brown K. Thomas L. Ferreira M. A. R. Bender D.. (2007). PLINK: a tool set for whole-genome association and population-based linkage analyses. Am. J. Hum. Genet. 81, 559–575. doi: 10.1086/519795, PMID: 17701901 PMC1950838

[ref69] Quinlan A. R. Hall I. M. (2010). BEDTools: a flexible suite of utilities for comparing genomic features. Bioinformatics 26, 841–842. doi: 10.1093/bioinformatics/btq033, PMID: 20110278 PMC2832824

[ref70] R Core Team (2014). R: A Language and Environment for Statistical Computing. Vienna, Australia: R Foundation for Statistical Computing.

[ref71] Rabbi I. Y. Hamblin M. T. Lava Kumar P. Gedil M. A. Ikpan A. S. Jannink J.-L.. (2014). High-resolution mapping of resistance to cassava mosaic geminiviruses in cassava using genotyping-by-sequencing and its implications for breeding. Virus Res. 186, 87–96. doi: 10.1016/j.virusres.2013.12.028, PMID: 24389096

[ref72] Rabbi I. Y. Kayondo S. I. Bauchet G. Yusuf M. Aghogho C. I. Ogunpaimo K.. (2020). Genome-wide association analysis reveals new insights into the genetic architecture of defensive, agro-morphological and quality-related traits in cassava. Plant Mol. Biol. doi: 10.1007/s11103-020-01038-3 [Epub ahead of print], PMID: 32734418 PMC9162993

[ref73] Rabbi I. Y. Udoh L. I. Wolfe M. D. Parkes E. Y. Gedil M. A. Dixon A. G. O.. (2017). Genome-wide association mapping of correlated traits in cassava: dry matter and total carotenoid content. Plant Genome 10, 1–14. doi: 10.3835/plantgenome2016.09.0094, PMID: 29293815 PMC7822061

[ref74] Raji A. A. Ladeinde T. A. O. Dixon A. G. O. (2007). Agronomic traits and tuber quality attributes of farmer grown cassava landraces in Nigeria. J. Trop. Agric. 45, 9–13.

[ref75] Ramu P. Esuma W. Kawuki R. S. Rabbi I. Y. Egesi C. N. Bredeson J. V.. (2017). Cassava haplotype map highlights fixation of deleterious mutations during clonal propagation. Nat. Genet. 49, 959–963. doi: 10.1038/ng.3845, PMID: 28416819

[ref76] Ranocha P. Denancé N. Vanholme R. Freydier A. Martinez Y. Hoffmann L.. (2010). Walls are thin 1 (WAT1), an Arabidopsis homolog of *Medicago truncatula NODULIN21*, is a tonoplast-localized protein required for secondary wall formation in fibers. Plant J. 63, 469–483. doi: 10.1111/j.1365-313X.2010.04256.x, PMID: 20497379

[ref77] Rosli H. G. Zheng Y. Pombo M. A. Zhong S. Bombarely A. Fei Z.. (2013). Transcriptomics-based screen for genes induced by flagellin and repressed by pathogen effectors identifies a cell wall-associated kinase involved in plant immunity. Genome Biol. 14:R139. doi: 10.1186/gb-2013-14-12-r139, PMID: 24359686 PMC4053735

[ref78] Ross H. A. McDougall G. J. Vincent J. F. V. Stewart D. Verrall S. Taylor M. A. (2010). Discerning intra-tuber differences in textural properties in cooked *Solanum tuberosum* group Tuberosum and group Phureja tubers. J. Sci. Food Agric. 90, 1527–1532. doi: 10.1002/jsfa.3979, PMID: 20549807

[ref79] Ross H. A. Morris W. L. Ducreux L. J. M. Hancock R. D. Verrall S. R. Morris J. A.. (2011a). Pectin engineering to modify product quality in potato. Plant Biotechnol. J. 9, 848–856. doi: 10.1111/j.1467-7652.2011.00591.x, PMID: 21281424

[ref80] Ross H. A. Wright K. M. McDougall G. J. Roberts A. G. Chapman S. N. Morris W. L.. (2011b). Potato tuber pectin structure is influenced by pectin methyl esterase activity and impacts on cooked potato texture. J. Exp. Bot. 62, 371–381. doi: 10.1093/jxb/erq280, PMID: 20855456 PMC2993920

[ref81] Simko I. Eujayl I. van Hintum T. J. L. (2012). Empirical evaluation of DArT, SNP, and SSR marker systems for genotyping, clustering, and assigning sugar beet hybrid varieties into populations. Plant Sci. 184, 54–62. doi: 10.1016/j.plantsci.2011.12.009, PMID: 22284710

[ref82] Smith D. L. Gross K. C. (2000). A family of at least seven β-galactosidase genes is expressed during tomato fruit development. Plant Physiol. 123, 1173–1183. doi: 10.1104/pp.123.3.1173, PMID: 10889266 PMC59080

[ref83] Song C. Zhao S. Hong X. Liu J. Schulenburg K. Schwab W. (2016). A UDP-glucosyltransferase functions in both acylphloroglucinol glucoside and anthocyanin biosynthesis in strawberry (Fragaria × ananassa). Plant J. 85, 730–742. doi: 10.1111/tpj.13140, PMID: 26859691

[ref84] Stracke R. Werber M. Weisshaar B. (2001). The R2R3-MYB gene family in *Arabidopsis thaliana*. Curr. Opin. Plant Biol. 4, 447–456. doi: 10.1016/S1369-5266(00)00199-0, PMID: 11597504

[ref85] Sukumaran S. Reynolds M. P. Sansaloni C. (2018). Genome-wide association analyses identify QTL hotspots for yield and component traits in durum wheat grown under yield potential, drought, and heat stress environments. Front. Plant Sci. 9:81. doi: 10.3389/fpls.2018.00081, PMID: 29467776 PMC5808252

[ref86] Tateishi A. Inoue H. Shiba H. Yamaki S. (2001). Molecular cloning of β-galactosidase from Japanese pear (*Pyrus pyrifolia*) and its gene expression with fruit ripening. Plant Cell Physiol. 42, 492–498. doi: 10.1093/pcp/pce059, PMID: 11382815

[ref87] Trainotti L. Spinello R. Piovan A. Spolaore S. Casadoro G. (2001). β-galactosidases with a lectin-like domain are expressed in strawberry. J. Exp. Bot. 52, 1635–1645. doi: 10.1093/jexbot/52.361.1635, PMID: 11479328

[ref88] Udoh L. I. Gedil M. Parkes E. Y. Kulakow P. Adesoye A. Nwuba C.. (2017). Candidate gene sequencing and validation of SNP markers linked to carotenoid content in cassava (*Manihot esculenta* Crantz). Mol. Breed. 37:123. doi: 10.1007/s11032-017-0718-5

[ref89] VanRaden P. M. (2008). Efficient methods to compute genomic predictions. J. Dairy Sci. 91, 4414–4423. doi: 10.3168/jds.2007-0980, PMID: 18946147

[ref90] Vazquez A. Bates D. Rosa G. Gianola D. Weigel K. (2010). Technical note: an R package for fitting generalized linear mixed models in animal breeding. J. Anim. Sci. 88, 497–504. doi: 10.2527/jas.2009-1952, PMID: 19820058

[ref91] Welsch R. Arango J. Bär C. Salazar B. Al-Babili S. Beltrán J.. (2010). Provitamin A accumulation in cassava (*Manihot esculenta*) roots driven by a single nucleotide polymorphism in a phytoene synthase gene. Plant Cell 22, 3348–3356. doi: 10.1105/tpc.110.077560, PMID: 20889914 PMC2990137

[ref92] Wolfe M. D. Rabbi I. Y. Egesi C. Hamblin M. Kawuki R. S. Kulakow P. A.. (2016). Genome-wide association and prediction reveals genetic architecture of cassava mosaic disease resistance and prospect for rapid genetic improvement. Plant Genome 9, 1–13. doi: 10.3835/plantgenome2015.11.0118, PMID: 27898832

[ref93] Yang H. Liu J. Dang M. Zhang B. Li H. Meng R.. (2018a). Analysis of β-galactosidase during fruit development and ripening in two different texture types of apple cultivars. Front. Plant Sci. 9:539. doi: 10.3389/fpls.2018.00539, PMID: 29740469 PMC5928752

[ref94] Yang B. Xu S. Xu L. You H. Xiang X. (2018b). Effects of Wx and its interaction with SSIII-2 on rice eating and cooking qualities. Front. Plant Sci. 9:456. doi: 10.3389/fpls.2018.00456, PMID: 29692791 PMC5902675

[ref95] Yu J. Pressoir G. Briggs W. H. Bi I. V. Yamasaki M. Doebley J. F.. (2006). A unified mixed-model method for association mapping that accounts for multiple levels of relatedness. Nat. Genet. 38, 203–208. doi: 10.1038/ng1702, PMID: 16380716

[ref96] Zhang S. Chen C. Li L. Meng L. Singh J. Jiang N.. (2005). Evolutionary expansion, gene structure, and expression of the rice wall-associated kinase gene family. Plant Physiol. 139, 1107–1124. doi: 10.1104/pp.105.069005, PMID: 16286450 PMC1283751

[ref97] Zhang Z. Ersoz E. Lai C. Q. Todhunter R. J. Tiwari H. K. Gore M. A.. (2010). Mixed linear model approach adapted for genome-wide association studies. Nat. Genet. 42, 355–360. doi: 10.1038/ng.546, PMID: 20208535 PMC2931336

[ref98] Zhang X. Zhang H. Li L. Lan H. Ren Z. Liu D.. (2016). Characterizing the population structure and genetic diversity of maize breeding germplasm in Southwest China using genome-wide SNP markers. BMC Genomics 17:697. doi: 10.1186/s12864-016-3041-3, PMID: 27581193 PMC5007717

[ref99] Zhao C. Hua L. N. Liu X. F. Li Y. Z. Shen Y. Y. Guo J. X. (2017). Sucrose synthase FaSS1 plays an important role in the regulation of strawberry fruit ripening. Plant Growth Regul. 81, 175–181. doi: 10.1007/s10725-016-0189-4

[ref100] Zuo W. Chao Q. Zhang N. Ye J. Tan G. Li B.. (2015). A maize wall-associated kinase confers quantitative resistance to head smut. Nat. Genet. 47, 151–157. doi: 10.1038/ng.3170, PMID: 25531751

