# Supplementary material for: Genome-Wide Association Study of Root Mealiness and Other Texture-Associated Traits in Cassava
Source: Front Plant Sci. 2021 Dec 17;12:770434. doi: 10.3389/fpls.2021.770434 (PMC8719520; doi:10.3389/fpls.2021.770434)
Supplement: Supplementary file 1 [file Data_Sheet_1.docx]

**Supplementary Table S1׀** List of cassava accessions used in the present study.

| Entry | Genotype | Code | Root flesh colour |  | Entry | Genotype | Code | Root flesh colour |  | Entry | Genotype | Code | Root flesh colour |
| --- | --- | --- | --- | --- | --- | --- | --- | --- | --- | --- | --- | --- | --- |
| 1 | AR14-4 | G1 | Cream |  | 26 | COB4-100 | G26 | Cream |  | 51 | NR050667 | G51 | White |
| 2 | AR1-45 | G2 | White |  | 27 | COB4-27 | G27 | Cream |  | 52 | NR060169 | G52 | Cream |
| 3 | AR1-82 | G3 | Yellow |  | 28 | COB4-77 | G28 | White |  | 53 | NR060242 | G53 | White |
| 4 | AR31-1 | G4 | Cream |  | 29 | COB5-01 | G29 | Yellow |  | 54 | NR060246 | G54 | White |
| 5 | AR9-45 | G5 | White |  | 30 | COB5-11 | G30 | White |  | 55 | NR060251 | G55 | Yellow |
| 6 | B1-19 | G6 | Yellow |  | 31 | COB5-17 | G31 | Yellow |  | 56 | NR060333 | G56 | Cream |
| 7 | B1-23 | G7 | Yellow |  | 32 | COB5-44 | G32 | Yellow |  | 57 | NR060789 | G57 | Cream |
| 8 | B1-25 | G8 | Yellow |  | 33 | COB5-86 | G33 | Yellow |  | 58 | NR070004 | G58 | Yellow |
| 9 | B1-26 | G9 | Cream |  | 34 | COB6-4 | G34 | Cream |  | 59 | NR070240 | G59 | Yellow |
| 10 | B1-29 | G10 | Yellow |  | 35 | COB7-197 | G35 | White |  | 60 | NR070632 | G60 | Yellow |
| 11 | B1-48 | G11 | Cream |  | 36 | CR14B-218 | G36 | Cream |  | 61 | NR090001 | G61 | White |
| 12 | B1-5 | G12 | Yellow |  | 37 | CR15B-3 | G37 | White |  | 62 | NR090088 | G62 | Cream |
| 13 | B1-50 | G13 | Cream |  | 38 | CR24-9 | G38 | Cream |  | 63 | NR090127 | G63 | Cream |
| 14 | B1-51 | G14 | Cream |  | 39 | CR35-10 | G39 | Cream |  | 64 | NR090142 | G64 | Yellow |
| 15 | B1-56 | G15 | Yellow |  | 40 | CR44-6 | G40 | Cream |  | 65 | NR090146 | G65 | White |
| 16 | B1-58 | G16 | Cream |  | 41 | CR528-26 | G41 | White |  | 66 | NR090162 | G66 | Yellow |
| 17 | B1-61 | G17 | Yellow |  | 42 | CR8A-22 | G42 | White |  | 67 | NR090176 | G67 | Cream |
| 18 | B1-67 | G18 | Yellow |  | 43 | IBA083739 | G43 | Yellow |  | 68 | NR090182 | G68 | Yellow |
| 19 | B1-78 | G19 | Cream |  | 44 | MM915280 | G44 | White |  | 69 | NR100018 | G69 | White |
| 20 | B1-95 | G20 | Yellow |  | 45 | MM961751 | G45 | Cream |  | 70 | NR100024 | G70 | White |
| 21 | B2-37 | G21 | Yellow |  | 46 | NR010161 | G46 | White |  | 71 | NR100077 | G71 | White |
| 22 | B4-6 | G22 | Yellow |  | 47 | NR010408 | G47 | Yellow |  | 72 | NR100106 | G72 | Yellow |
| 23 | B5-15 | G23 | Cream |  | 48 | NR050080 | G48 | Yellow |  | 73 | NR100112 | G73 | Cream |
| 24 | B5-19 | G24 | Yellow |  | 49 | NR050166 | G49 | Cream |  | 74 | NR100126 | G74 | White |
| 25 | COB1-163 | G25 | Cream |  | 50 | NR050362 | G50 | White |  | 75 | NR100196 | G75 | White |

**Supplementary Table S1׀** Continued

| Entry | Genotype | Code | Root flesh colour |  | Entry | Genotype | Code | Root flesh colour |  | Entry | Genotype | Code | Root flesh colour |
| --- | --- | --- | --- | --- | --- | --- | --- | --- | --- | --- | --- | --- | --- |
| 76 | NR100216 | G76 | White |  | 101 | NR110213 | G101 | White |  | 126 | NR1S1185 | G126 | Yellow |
| 77 | NR100225 | G77 | Cream |  | 102 | NR110223 | G102 | Cream |  | 127 | TME419 | G127 | White |
| 78 | NR100248 | G78 | Yellow |  | 103 | NR110228 | G103 | Cream |  | 128 | TMEB693 | G128 | White |
| 79 | NR100252 | G79 | Yellow |  | 104 | NR110232 | G104 | Cream |  | 129 | TMS010085 | G129 | Cream |
| 80 | NR100265 | G80 | White |  | 105 | NR110238 | G105 | Cream |  | 130 | TMS0101134 | G130 | Cream |
| 81 | NR100297 | G81 | White |  | 106 | NR110267 | G106 | Cream |  | 131 | TMS010169 | G131 | White |
| 82 | NR100325 | G82 | White |  | 107 | NR110270 | G107 | Cream |  | 132 | TMS010354 | G132 | White |
| 83 | NR100401 | G83 | Cream |  | 108 | NR110315 | G108 | White |  | 133 | TMS011097 | G133 | Yellow |
| 84 | NR100417 | G84 | Cream |  | 109 | NR110337 | G109 | Cream |  | 134 | TMS011368 | G134 | Yellow |
| 85 | NR100449 | G85 | Cream |  | 110 | NR110348 | G110 | White |  | 135 | TMS011412 | G135 | Yellow |
| 86 | NR100450 | G86 | Yellow |  | 111 | NR110372 | G111 | Yellow |  | 136 | TMS050128 | G136 | Yellow |
| 87 | NR100499 | G87 | Cream |  | 112 | NR110376 | G112 | Cream |  | 137 | TMS0501653 | G137 | Yellow |
| 88 | NR110031 | G88 | Yellow |  | 113 | NR110411 | G113 | Yellow |  | 138 | TMS050311 | G138 | Yellow |
| 89 | NR110044 | G89 | Yellow |  | 114 | NR110433 | G114 | Cream |  | 139 | TMS050540 | G139 | Cream |
| 90 | NR110079 | G90 | Cream |  | 115 | NR110439 | G115 | Yellow |  | 140 | TMS050752 | G140 | Yellow |
| 91 | NR110084 | G91 | Cream |  | 116 | NR110476 | G116 | White |  | 141 | TMS051570 | G141 | Yellow |
| 92 | NR110109 | G92 | Cream |  | 117 | NR110485 | G117 | White |  | 142 | TMS051600 | G142 | Yellow |
| 93 | NR110118 | G93 | White |  | 118 | NR110489 | G118 | Yellow |  | 143 | TMS051601 | G143 | Yellow |
| 94 | NR110160 | G94 | Cream |  | 119 | NR110490 | G119 | Cream |  | 144 | TMS051625 | G144 | Yellow |
| 95 | NR110165 | G95 | White |  | 120 | NR110512 | G120 | White |  | 145 | TMS070489 | G145 | Yellow |
| 96 | NR110169 | G96 | White |  | 121 | NR1S10064 | G121 | Cream |  | 146 | TMS070539 | G146 | Yellow |
| 97 | NR110176 | G97 | Cream |  | 122 | NR1S10097 | G122 | Cream |  | 147 | TMS070649 | G147 | Yellow |
| 98 | NR110178 | G98 | White |  | 123 | NR1S1018 | G123 | Cream |  | 148 | TMS30572 | G148 | White |
| 99 | NR110179 | G99 | Cream |  | 124 | NR1S1048 | G124 | Cream |  | 149 | TMS950211 | G149 | Yellow |
| 100 | NR110181 | G100 | Cream |  | 125 | NR1S1112 | G125 | Yellow |  | 150 | TMS961708 | G150 | White |


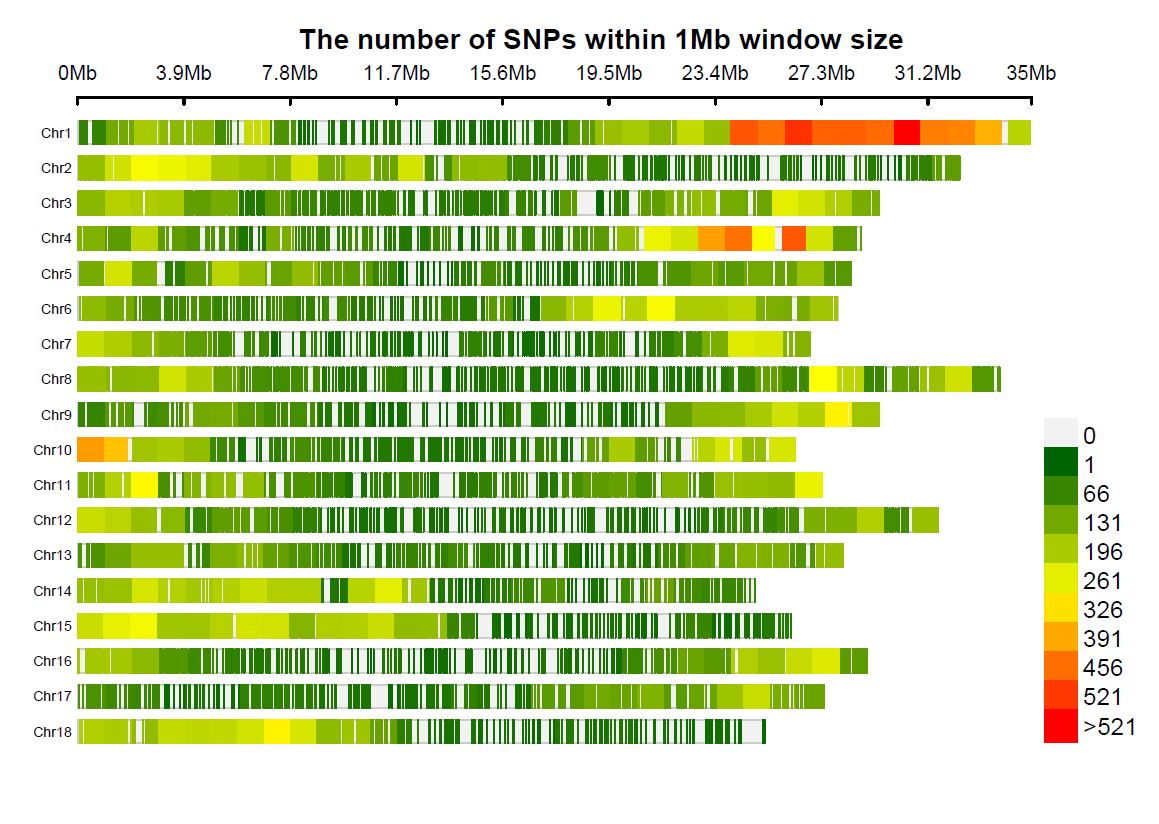


2, 227

2, 859

**No of SNPS**

2, 488

3, 401

3, 249

3, 321

2, 375

3, 469

3, 287

2, 783

3, 274

2, 581

2, 626

4, 324

3, 390

3, 871

7, 382

2, 885

**Supplementary Figure S1** **׀** Genome-wide SNP distributions on 18 chromosomes of cassava. The horizontal axis displays the chromosome length in base pairs; 0-521 colour-coded legend insert indicates the density of SNPs; number of SNPs is also indicated for each of the 18 chromosomes.

**Supplementary Table S2 ׀** Summary statistics of the 59,792 high quality SNP markers.

| Diversity parameter | Minimum | Maximum | Mean |
| --- | --- | --- | --- |
| MAF^a^ | 0.05 | 0.50 | 0.23 |
| Ho^b^ | 0.01 | 0.97 | 0.31 |
| He^c^ | 0.10 | 0.50 | 0.32 |
| PIC^d^ | 0.09 | 0.38 | 0.26 |
| Gene diversity | 0.10 | 0.50 | 0.32 |

^a^Minor allele frequency; ^b^Observed heterozygosity; ^c^Expected heterozygosity; ^d^Polymorphic information content.

**Supplementary Table S3 ׀** Summary of SNPs with genome-wide association significance for root mealiness and other textural attributes of boiled cassava roots.

| Trait | SNP_ID | Chr^a^ | Position | Allele | | MAF^b^ | *P* value | R^2c^ |
| --- | --- | --- | --- | --- | --- | --- | --- | --- |
|  |  |  |  | Major | Minor |  |  |  |
| Mealiness | S13_25716244 | 13 | 25716244 | C | G | 0.50 | 1.65E-05 | 0.18 |
| Mealiness | S13_25667749 | 13 | 25667749 | C | T | 0.49 | 2.02E-05 | 0.18 |
| Mealiness | S13_25591824 | 13 | 25591824 | T | C | 0.49 | 2.81E-05 | 0.17 |
| Mealiness | S13_25417306 | 13 | 25417306 | C | A | 0.48 | 3.08E-05 | 0.17 |
| Mealiness | S13_25394035 | 13 | 25394035 | C | T | 0.47 | 5.01E-05 | 0.17 |
| Mealiness | S6_319697 | 6 | 319697 | T | A | 0.06 | 6.31E-05 | 0.16 |
| Mealiness | S13_25447531 | 13 | 25447531 | A | C | 0.48 | 8.56E-05 | 0.16 |
| ADH^d^ | S18_3604145 | 18 | 3604145 | G | C | 0.14 | 1.41E-06 | 0.21 |
| ADH | S18_3573999 | 18 | 3573999 | A | C | 0.13 | 2.46E-06 | 0.20 |
| ADH | S18_3563031 | 18 | 3563031 | T | G | 0.15 | 5.27E-06 | 0.18 |
| ADH | S18_3414606 | 18 | 3414606 | C | G | 0.13 | 7.44E-06 | 0.18 |
| ADH | S18_3414613 | 18 | 3414613 | C | G | 0.13 | 7.44E-06 | 0.18 |
| ADH | S18_3297837 | 18 | 3297837 | T | G | 0.19 | 8.06E-06 | 0.18 |
| ADH | S18_3407893 | 18 | 3407893 | T | C | 0.13 | 1.00E-05 | 0.17 |
| ADH | S18_3342278 | 18 | 3342278 | A | C | 0.12 | 1.09E-05 | 0.17 |
| ADH | S18_3546520 | 18 | 3546520 | C | G | 0.12 | 1.13E-05 | 0.17 |
| ADH | S18_3549117 | 18 | 3549117 | G | T | 0.12 | 1.13E-05 | 0.17 |
| ADH | S18_3359436 | 18 | 3359436 | A | T | 0.20 | 1.16E-05 | 0.17 |
| ADH | S18_3215650 | 18 | 3215650 | T | A | 0.12 | 1.21E-05 | 0.17 |
| ADH | S18_3234621 | 18 | 3234621 | G | T | 0.12 | 1.21E-05 | 0.17 |
| ADH | S18_3237636 | 18 | 3237636 | A | G | 0.12 | 1.21E-05 | 0.17 |
| ADH | S18_3254073 | 18 | 3254073 | G | C | 0.12 | 1.21E-05 | 0.17 |
| ADH | S18_3540608 | 18 | 3540608 | C | A | 0.12 | 1.21E-05 | 0.17 |
| ADH | S18_3215390 | 18 | 3215390 | T | A | 0.13 | 1.23E-05 | 0.17 |
| ADH | S18_3327344 | 18 | 3327344 | T | A | 0.12 | 1.42E-05 | 0.17 |
| ADH | S18_3340515 | 18 | 3340515 | C | T | 0.12 | 1.42E-05 | 0.17 |
| ADH | S18_3340717 | 18 | 3340717 | A | G | 0.12 | 1.42E-05 | 0.17 |
| ADH | S18_3342049 | 18 | 3342049 | C | G | 0.12 | 1.42E-05 | 0.17 |
|  |  |  |  |  |  |  |  |  |

**Supplementary Table S3 ׀** Continued

| Trait | SNP_ID | Chr^a^ | Position | Allele | | MAF^b^ | *P* value | R^2c^ |
| --- | --- | --- | --- | --- | --- | --- | --- | --- |
|  |  |  |  | Major | Minor |  |  |  |
| ADH^d^ | S18_3604331 | 18 | 3604331 | T | A | 0.12 | 1.57E-05 | 0.17 |
| ADH | S18_3173420 | 18 | 3173420 | C | T | 0.12 | 1.59E-05 | 0.17 |
| ADH | S18_3241826 | 18 | 3241826 | C | G | 0.14 | 1.66E-05 | 0.17 |
| ADH | S18_3215371 | 18 | 3215371 | A | G | 0.13 | 1.67E-05 | 0.17 |
| ADH | S18_3544492 | 18 | 3544492 | G | C | 0.13 | 1.70E-05 | 0.17 |
| ADH | S18_3309923 | 18 | 3309923 | C | G | 0.18 | 1.72E-05 | 0.17 |
| ADH | S18_3170754 | 18 | 3170754 | T | C | 0.12 | 1.91E-05 | 0.16 |
| ADH | S18_3170789 | 18 | 3170789 | T | C | 0.12 | 1.91E-05 | 0.16 |
| ADH | S18_2907306 | 18 | 2907306 | G | A | 0.12 | 2.04E-05 | 0.16 |
| ADH | S18_3549464 | 18 | 3549464 | T | A | 0.13 | 2.11E-05 | 0.16 |
| ADH | S18_3645853 | 18 | 3645853 | C | T | 0.12 | 2.36E-05 | 0.16 |
| ADH | S18_3266976 | 18 | 3266976 | C | G | 0.11 | 2.44E-05 | 0.16 |
| ADH | S18_3347163 | 18 | 3347163 | T | C | 0.17 | 2.54E-05 | 0.16 |
| ADH | S18_3347197 | 18 | 3347197 | T | C | 0.17 | 2.54E-05 | 0.16 |
| ADH | S18_3539037 | 18 | 3539037 | T | C | 0.11 | 2.93E-05 | 0.16 |
| ADH | S18_3421276 | 18 | 3421276 | G | A | 0.12 | 3.34E-05 | 0.16 |
| ADH | S18_3430536 | 18 | 3430536 | C | T | 0.12 | 3.34E-05 | 0.16 |
| ADH | S18_3523503 | 18 | 3523503 | A | T | 0.12 | 3.34E-05 | 0.16 |
| ADH | S18_3282502 | 18 | 3282502 | T | C | 0.12 | 3.35E-05 | 0.15 |
| ADH | S18_3108177 | 18 | 3108177 | T | C | 0.15 | 3.50E-05 | 0.15 |
| ADH | S18_3506350 | 18 | 3506350 | A | G | 0.12 | 3.64E-05 | 0.15 |
| ADH | S18_3253429 | 18 | 3253429 | A | G | 0.12 | 3.64E-05 | 0.15 |
| ADH | S18_3512011 | 18 | 3512011 | T | C | 0.12 | 3.90E-05 | 0.15 |
| ADH | S18_3320510 | 18 | 3320510 | G | C | 0.24 | 4.24E-05 | 0.15 |
| ADH | S18_3183508 | 18 | 3183508 | T | A | 0.11 | 4.57E-05 | 0.15 |
| ADH | S18_2594316 | 18 | 2594316 | G | A | 0.10 | 5.40E-05 | 0.15 |
| ADH | S18_3196932 | 18 | 3196932 | C | T | 0.13 | 5.63E-05 | 0.15 |
| ADH | S18_3237606 | 18 | 3237606 | A | C | 0.13 | 5.63E-05 | 0.15 |
| ADH | S18_3153983 | 18 | 3153983 | C | T | 0.11 | 6.07E-05 | 0.15 |
| ADH | S18_3360375 | 18 | 3360375 | T | C | 0.11 | 6.53E-05 | 0.14 |
| ADH | S18_3399451 | 18 | 3399451 | A | T | 0.12 | 6.80E-05 | 0.14 |
| ADH | S18_3506169 | 18 | 3506169 | G | C | 0.11 | 7.78E-05 | 0.14 |
| ADH | S18_3506192 | 18 | 3506192 | T | A | 0.11 | 7.78E-05 | 0.14 |
| ADH | S18_3506207 | 18 | 3506207 | A | G | 0.11 | 7.78E-05 | 0.14 |
| ADH | S18_2946161 | 18 | 2946161 | A | C | 0.11 | 7.83E-05 | 0.14 |
| ADH | S18_3024011 | 18 | 3024011 | T | A | 0.11 | 7.83E-05 | 0.14 |
| ADH | S18_3102263 | 18 | 3102263 | A | T | 0.11 | 7.83E-05 | 0.14 |
|  |  |  |  |  |  |  |  |  |

**Supplementary Table S3 ׀** Continued

| Trait | SNP_ID | Chr^a^ | Position | Allele | | MAF^b^ | *P* value | R^2c^ |
| --- | --- | --- | --- | --- | --- | --- | --- | --- |
|  |  |  |  | Major | Minor |  |  |  |
| ADH^d^ | S18_3266678 | 18 | 3266678 | G | T | 0.13 | 8.12E-05 | 0.14 |
| ADH | S18_3279394 | 18 | 3279394 | G | A | 0.18 | 8.60E-05 | 0.14 |
| ADH | S18_2556956 | 18 | 2556956 | C | G | 0.11 | 8.89E-05 | 0.14 |
| ADH | S18_2556981 | 18 | 2556981 | T | C | 0.11 | 8.89E-05 | 0.14 |
| Aroma | S17_7460784 | 17 | 7460784 | C | T | 0.11 | 9.90E-05 | 0.12 |
| Colour | S1_25733720 | 1 | 25733720 | T | C | 0.44 | 9.05E-05 | 0.18 |
| Colour | S1_25695204 | 1 | 25695204 | A | G | 0.49 | 9.85E-05 | 0.18 |
| Colour | S1_25695218 | 1 | 25695218 | G | T | 0.49 | 9.85E-05 | 0.18 |
| Fibre | S5_21198714 | 5 | 21198714 | C | T | 0.08 | 9.84E-05 | 0.16 |
| Firmness | S4_23643811 | 4 | 23643811 | A | G | 0.43 | 1.08E-05 | 0.17 |
| Taste | S10_3499386 | 10 | 3499386 | A | G | 0.24 | 5.02E-05 | 0.15 |
| Taste | S1_24825469 | 1 | 24825469 | C | A | 0.49 | 5.32E-05 | 0.15 |
| Taste | S10_3326655 | 10 | 3326655 | A | T | 0.24 | 6.42E-05 | 0.14 |
| Taste | S10_3444639 | 10 | 3444639 | G | T | 0.24 | 7.98E-05 | 0.14 |
| Taste | S10_3290580 | 10 | 3290580 | C | T | 0.27 | 9.07E-05 | 0.14 |
|  |  |  |  |  |  |  |  |  |

^a^Chromosome; ^b^Minor allele frequency; ^c^Proportion of phenotypic variation explained by SNPs; ^d^Adhesiveness.
